# Genomic profiling of dioecious *Amaranthus* species provides novel insights into species relatedness and sex genes

**DOI:** 10.1186/s12915-023-01539-9

**Published:** 2023-02-20

**Authors:** Damilola A. Raiyemo, Lucas K. Bobadilla, Patrick J. Tranel

**Affiliations:** Department of Crop Sciences, University of Illinois, Urbana, IL 61801 USA

**Keywords:** Comparative genomics, Genome evolution, *Amaranthus* species, Dioecious amaranths, Male-specific Y region, Sex determination

## Abstract

**Background:**

*Amaranthus* L. is a diverse genus consisting of domesticated, weedy, and non-invasive species distributed around the world. Nine species are dioecious, of which *Amaranthus palmeri* S. Watson and *Amaranthus tuberculatus* (Moq.) J.D. Sauer are troublesome weeds of agronomic crops in the USA and elsewhere. Shallow relationships among the dioecious *Amaranthus* species and the conservation of candidate genes within previously identified *A. palmeri* and *A. tuberculatus* male-specific regions of the Y (MSYs) in other dioecious species are poorly understood. In this study, seven genomes of dioecious amaranths were obtained by paired-end short-read sequencing and combined with short reads of seventeen species in the family Amaranthaceae from NCBI database. The species were phylogenomically analyzed to understand their relatedness. Genome characteristics for the dioecious species were evaluated and coverage analysis was used to investigate the conservation of sequences within the MSY regions.

**Results:**

We provide genome size, heterozygosity, and ploidy level inference for seven newly sequenced dioecious *Amaranthus* species and two additional dioecious species from the NCBI database. We report a pattern of transposable element proliferation in the species, in which seven species had more *Ty3* elements than *copia* elements while *A. palmeri* and *A. watsonii* had more *copia* elements than *Ty3* elements, similar to the TE pattern in some monoecious amaranths. Using a Mash-based phylogenomic analysis, we accurately recovered taxonomic relationships among the dioecious *Amaranthus* species that were previously identified based on comparative morphology. Coverage analysis revealed eleven candidate gene models within the *A. palmeri* MSY region with male-enriched coverages, as well as regions on scaffold 19 with female-enriched coverage, based on *A. watsonii* read alignments. A previously reported *FLOWERING LOCUS T* (*FT*) within *A. tuberculatus* MSY contig was also found to exhibit male-enriched coverages for three species closely related to *A. tuberculatus* but not for *A. watsonii* reads. Additional characterization of the *A. palmeri* MSY region revealed that 78% of the region is made of repetitive elements, typical of a sex determination region with reduced recombination.

**Conclusions:**

The results of this study further increase our understanding of the relationships among the dioecious species of the *Amaranthus* genus as well as revealed genes with potential roles in sex function in the species.

**Supplementary Information:**

The online version contains supplementary material available at 10.1186/s12915-023-01539-9.

## Background

The genus *Amaranthus* L. is a diverse plant group of 70–80 species distributed across the world’s temperate and tropical regions [1]. Nine of these species [*Amaranthus acanthochiton* J.D. Sauer, *Amaranthus arenicola* I.M. Johnson, *Amaranthus australis* (A. Gray) J.D. Sauer, *Amaranthus cannabinus* (L.) J.D. Sauer, *Amaranthus floridanus* (S. Watson) J.D. Sauer, *Amaranthus tuberculatus* (Moq.) J.D. Sauer, *Amaranthus greggii* S. Watson, *Amaranthus watsonii* Standley, and *Amaranthus palmeri* S. Watson] are dioecious (i.e., separate male and female individual plants), native to North America and grouped collectively into the subgenus *Acnida* (L.) Aellen ex K.R. Robertson [2–4].

The *Amaranthus* genus has been described as taxonomically challenging due to morphological similarities among species [5]. Relationships among species of the genus, including the dioecious ones, were previously investigated using several molecular markers and phylogenetic frameworks [6–10]. Stetter and Schmid [9], with an objective to elucidate the domestication history of cultivated amaranths, used genotyping-by-sequencing (GBS) for 35 species of the genus in neighbor joining and multispecies coalescent (MSC) frameworks to infer *A. hybridus* as likely ancestor of the cultivated amaranths, *A. caudatus*, *A. cruentus*, and *A. hypochondriacus*. In the most recent attempt to reconstruct the evolutionary relationships among the species of the genus, Waselkov et al. [10] sampled 58 species, including the nine dioecious species, and used six molecular markers (ITS, *A36*, *G3PDH*, *waxy*, *trnL5’*-*trnL3’*, and *matk/trnK*) in a maximum parsimony and Bayesian inference phylogenetic framework. Trees from both studies were congruent with high support for deeper node relationships, such as species clustering or clades corresponding to previously defined three subgenera, *Acnida*, *Amaranthus* and *Albersia* [4]. Relationships among the dioecious species along “shallow” nodes however were poorly resolved with weak supports and, thus, some relationships remain unclear (e.g., is *A. tuberculatus* more closely related to *A. arenicola* than to *A. floridanus*?).

While advances in molecular phylogenetics have increased the level of inference we can draw on trait evolution or species relationships, poorly resolved trees resulting from biological processes (e.g., ancient or recent hybridization, incomplete lineage sorting, introgression or rapid radiation) or systematic errors (e.g., low parsimony-informativeness of markers) still make inference on trait evolution intractable for some genera [11]. Several methods estimating phylogenetic relationships that put into consideration these biological processes have gained attention [12–14]; however, few are able to explicitly estimate species trees from phylogenomic data taking into account several sources of conflict and heterogeneity in molecular substitution [15]. Thus, complementary approaches are often required for robust relationship inference. Phylogenetic approaches (e.g., *k*-mer-based method) that by-pass challenges inherent in alignment- or assembly-based methods have been proposed, offering flexibility to sequence analysis and better use of computing power compared to alignment-based methods [16, 17]. For instance, the MinHash algorithm [18] was implemented in a sequence clustering tool, Mash [19], and among 74 alignment-free (AF) methods, Mash was shown to have the highest performance for genome-based phylogeny of plants using unassembled reads [20].

Aside from interests in the evolutionary relationship among *Amaranthus* species, there is also renewed interests in the dioecious species for their weedy trait characteristics [21, 22] and their mechanisms of sex determination or dioecy evolution [23, 24]. Although many of the dioecious species are restricted to their geographic range and currently of little economic importance with regards to food source relative to cultivated monoecious species [1, 25, 26], *Amaranthus tuberculatus* and *A. palmeri* are two agronomically important weeds in North America [27] and have been the focus of many research studies [22, 28–31]. The dioecious nature of both species ensures obligate outcrossing, thus enhancing high genetic diversity, prolific seed production, rapid adaptation, and spread of herbicide resistance [21, 22, 32, 33]. While dioecy confers evolutionary advantages [34, 35], a disadvantage, however, believed to be taking place naturally, is that bottleneck events could result in populations that are depleted of one of the two sexes, and if not for sex reversion, the population would collapse and thus become locally extinct [36]. Considering this disadvantage an advantage from a weed management standpoint, artificial gender manipulation, whereby sex ratios could be biased towards one gender and the genetic factors involved are inherited in a non-Mendelian pattern via a gene drive system, was proposed as a possible strategy for management of weedy dioecious *Amaranthus* species [37, 38].

Only until recently have the genes and the mechanisms involved in sex determination been elucidated for a few plant species [39–45]. For the amaranths, previous work on dioecy confirmed males of *A. tuberculatus* and *A. palmeri* are heterogametic and, thus, have an XY sex chromosome system [23, 46]. The male-specific region of the Y (MSY) for both species were subsequently identified, spanning a ~ 1.3-Mb region with 121 gene models for *A. palmeri* while several contigs with a total length of 4.6 Mb and containing 147 gene models were identified for the *A. tuberculatus* MSY region [23, 24, 47]. Lack of synteny between the MSY regions of both species [23, 24], and the clustering of *A. palmeri* with monoecious species in the nuclear tree from Waselkov et al.’s phylogeny [10], led Montgomery et al. [24] to infer that the two species likely evolved dioecy independently. However, the chloroplast tree from the same study that generated the nuclear tree showed a single monophyletic clade for the dioecious *Amaranthus* species [10]. Simultaneously, Neves et al. [23] also demonstrated that dioecy in both *A. palmeri* and *A. tuberculatus* could be under the control of separate genomic regions. Based on the above evidence, we hypothesize two origins of dioecy: one shared by *A. palmeri* and *A. watsonii* and another shared by the remaining dioecious amaranths [29]. While male-specific regions in closely related species could differ in size or content, there is evidence that the same gene(s) or dioecy mechanism could still be recruited across the species [42].

The objective of this research was to use comparative genomics to investigate dioecy within the *Amaranthus* genus. We obtained whole-genome sequence from seven dioecious amaranths, and report genome characteristics, transposable element (TE) proliferation patterns, and phylogenomic relationships among the species. We identified genomic regions including candidate genes within *A. palmeri* and *A. tuberculatus* MSY region that exhibit male-enriched coverages across other dioecious *Amaranthus* species and could have roles in sex function. Finally, we elucidated repeat contents for the *A. palmeri* MSY region to test the hypothesis that typical sex determination regions have suppressed recombination and accumulate repetitive sequences [48–50].

## Results

### Genome size, heterozygosity, and ploidy estimation

**Fig. 1:**
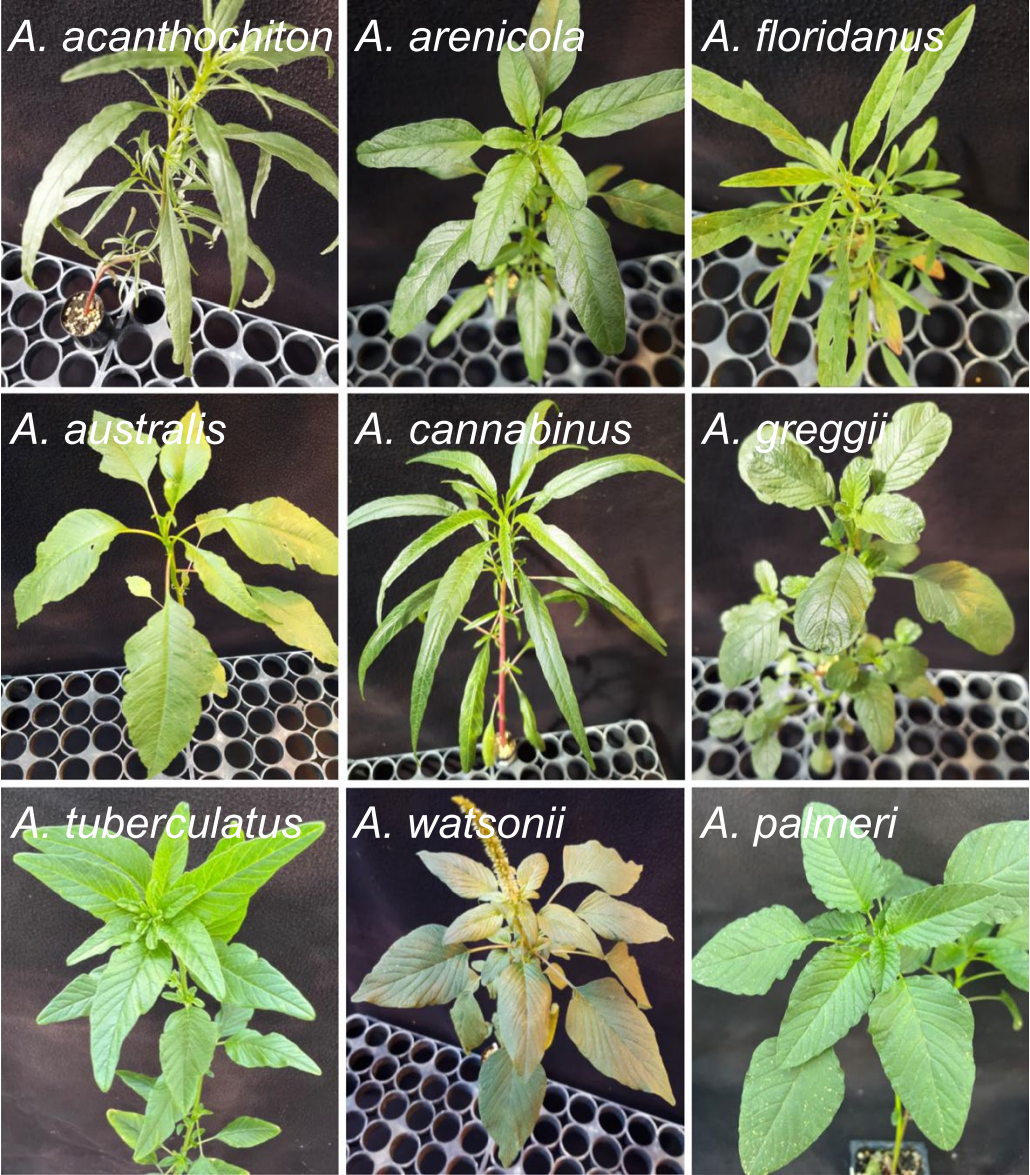
Representative individuals of the nine dioecious *Amaranthus* species

We employed *k*-mer-based tools to estimate genome sizes, heterozygosity, and ploidy for dioecious amaranths (Fig. 1). Estimates of genome sizes using GenomeScope [51] were 793.3 Mb (*A. australis*), 702.0 Mb (*A. cannabinus*), 684.6 Mb (*A. greggii*), 621.5 Mb (*A. acanthochiton*), 615.8 Mb (*A. tuberculatus*), 596.6 Mb (*A. floridanus*), 563.1 Mb (*A. arenicola*), 399.9 Mb (*A. watsonii*), and 374.4 Mb (*A. palmeri*) (Additional file 2: Fig. S1 – S9). The genome size estimates fall within the confidence bounds of previously reported genome sizes for *A. australis* (95% CI 735.7–912.8), *A. floridanus* (95% CI 543.5–772.9), and *A. palmeri* (95% CI 307.1–536.5) based on flow cytometry while the estimate for *A. tuberculatus* was 5.6 Mb lower than the lower confidence limit from previous estimate (95% CI 621.4–729.8) [9]. Analysis of raw reads of monoecious species (*A. hybridus* SRR12075659, *A. hypochondriacus* SRR2106212, and *A. cruentus* SRR13980261) also revealed genome size estimates consistent with previous flow cytometry results (Additional file 2: Fig. S10 – S12). The estimate of 398 Mb for *A. cruentus* reported in Ma et al. [52] however appears to be underestimated based on our reanalysis. We report a genome size of 489 Mb for the species which is consistent with previous estimates from flow cytometry (Additional file 2: Fig. S12).

CovEST repeats model and FindGSE yielded apparent overestimations of genome sizes for all species while CovEST basic model gave both apparent over and underestimates (Additional file 3: Table S1). CovEST and FindGSE, like GenomeScope, estimate genome characteristics from *k*-mer frequencies; however, they differ in the distribution or models adopted. GenomeScope fits a non-linear least square to a negative binomial distribution using Levenberg–Marquardt algorithm [51], CovEST use a Poisson distribution for *k*-mer abundance spectrum adopting a probabilistic framework [53], and FindGSE fits *k*-mer frequencies with a skew normal distribution [54]. It is possible that the distribution or model adopted in fitting *k*-mer frequencies by CovEST or FindGSE is less suitable considering our *k*-mer count data*,* thereby resulting in the inflation of genome sizes. Similar observation where estimates from CovEST Repeat model was higher than estimates from GenomeScope were reported for species of beetles [55].

Estimates of heterozygosity for *A. palmeri* (2.72%), *A. watsonii* (2.07%), and *A. arenicola* (2.06%) were higher than those of the other species, which ranged from 0.03% for *A. cruentus* to 1.97% for *A. acanthochiton* (Additional file 2: Fig. S1 – S12), indicating that high allelic variation could introduce assembly difficulty for some of the species [56].

We predicted the ploidy level for each of the genomes using Smudgeplot [51] in order to determine if species were polyploids, which may impact downstream analysis (e.g., reads mapping). All seven of the dioecious species sequenced from this study, including two other dioecious species and monoecious ones, were inferred as diploids. The *k*-mer coverage (kcov) in GenomeScope plots also corresponds to the haploid *k*-mer coverage (1n) in Smudgeplot, indicating the accuracy of ploidy prediction (Additional file 2: Fig. S1 – S12). Smudgeplot initially inferred tetraploidy for *Amaranthus greggii* when it was allowed to automatically detect haploid *k*-mer coverage at 44, similar to when Smudgeplot originally predicted tetraploidy for the diploid *Fragaria iinumae* strawberry genome [51]. However, rerunning Smudgeplot with the *k*-mer coverage from GenomeScope (kcov = 42) and increasing the lower *k*-mer coverage threshold value, L, to 20 caused it to infer diploidy (Additional file 2: Fig. S7). Nevertheless, the proportion of “AABB” smudge was as high as “AB” smudge for *A. greggii* relative to other species, indicating higher rates of duplications or paralogs (Additional file 2: Fig. S7).

### Transposable element analysis of unassembled *Amaranthus* genomes

To gain insight into the impact of repetitive elements on genome structure of dioecious *Amaranthus* species, we subjected subsampled read pairs of the nine dioecious species to RepeatExplorer2 [57], a graph-based repetitive sequence clustering and characterization tool for Illumina raw reads. Subsampled reads correspond to 0.3X coverage for each genome (see “Methods”). Results of the repeat analysis are presented in Additional file 3: Table S2A.

The total TE content identified in the nine genomes of the dioecious amaranths in RepeatExplorer2 pipeline was less than the total TE content discovered in the genome assemblies of the species, *A. hypochondriacus* at 51.76% [58], *A. cruentus* at 57.7% [52], *A. hybridus* at 57.34%, *A. palmeri* at 56.03%, or *A. tuberculatus* at 66.06% (Additional file 3: Table S3 – S4). The total composition of TE for *A. tuberculatus* male genome reported here is similar to the 66.28% reported for a previously assembled female genome of the same species [59]. It is worth mentioning that 57.68% of *A. hypochondriacus* genome [9.49% *copia* and 7.88% *Ty3*] was made up of repetitive elements when the genome was reanalyzed using more recent TE discovery tools (Additional file 3: Table S3). A similar observation was reported for the human genome, where RepeatMasker identified 48% of the genome as TEs, a proportion that further increased to 53% on reanalysis of the genome with the addition of Dfam2.0 database [60].

Reanalysis of the short reads with dnaPipeTE pipeline and using a species-specific library from *A. hypochondriacus* identified more proportion of total TEs in the genomes (Additional file 3: Table S2B). Although both dnaPipeTE and RepeatExplorer2 operate on the same principle, dnaPipeTE could annotate a larger fraction of TEs [61]. Our analysis identified the abundance of low copy repeats as a major source of discrepancies between dnaPipeTE and RepeatExplorer2 repeat quantification for the amaranths (Additional file 3: Table S2B, Additional file 4: Fig. S1 – S12). The total TE estimates for *A. tuberculatus* and *A. hybridus* using dnaPipeTE were 10% less than the total TE in their genome assemblies (Additional file 3: Table S3). For *A. palmeri*, *A. hypochondriacus*, and *A. cruentus*, differences in total TE between dnaPipeTE and the genome assembly were 19%, 18%, and 22%, respectively.

Despite TEs being underestimated in our study, the dynamics of relative TE accumulation for species within the genus is still interesting. *Amaranthus acanthochiton*, *A. arenicola*, *A. australis*, *A. cannabinus*, *A. floridanus*, *A. tuberculatus*, and *A. greggii* had more *Ty3* element than *copia* element (Additional file 3: Table S3). This pattern of relative TE composition using raw reads of *Amaranthus tuberculatus* [6.62% *copia* and 8.29% *Ty3*] is similar to TE composition in its assembled genome, where *copia* elements made up 12.58% while *Ty3* elements made up 17.01% of the genome (Additional file 3: Table S3). *Amaranthus watsonii*, however, had more *copia* (4.11%) than *Ty3* elements (2.71%), similar to *A. palmeri* (3.46% *copia* and 2.64% *Ty3*). The pattern of LTR composition in the unassembled raw reads of *A. palmeri* is also similar to its genome assembly (9.73% *copia* and 7.79% *Ty3*) (Additional file 3: Table S4) and to assembly of other monoecious species, *A. hybridus* (9.32% *copia* and 8.66% *Ty3*; Additional file 3: Table S3), *A. cruentus* [13.9% *copia* and 10.5% *Ty3*; Ma et al. [52]], or *A. hypochondriacus* [6.93% *copia* and 4.81% *Ty3*; Lightfoot et al. [58]]. DnaPipeTE, like Repeatexplorer2, also estimated slightly more total repeat composition for *A. cannabinus* than *A. australis* despite our previous genome size estimation indicating *A. australis* genome is larger than that of *A. cannabinus*. Both species however had the highest genome sizes and highest total TE discovered relative to other dioecious species (Additional file 3: Table S2).

### Mash-based phylogenomic analysis

**Fig. 2:**
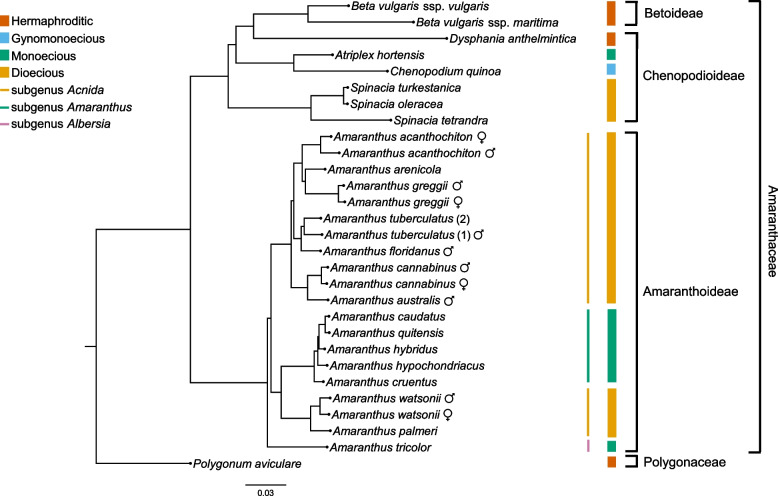
A Mash-based phylogeny using Illumina raw reads of dioecious *Amaranthus* species and other species of the Amaranthaceae family. *Polygonum aviculare* was used as outgroup

Considering the inconsistent tree topologies observed in previous phylogenetic studies of *Amaranthus* genus, and to avoid phylogenetic errors or noise that could result from assembling short reads, we investigated relatedness among the sequenced *Amaranthus* genomes and other members of the order Caryophyllales using an assembly- or alignment-free *k*-mer approach implemented in Mashtree [62]. As expected, sequenced females from four species included in the tree construction grouped together with their respective males (Fig. 2). Our analysis of genome relatedness showed species clustering corresponding to the three subgenera: *Acnida*, *Amaranthus*, and *Albersia* (Fig. 2), previously recognized based on fruit, bract, and tepal characteristics of pistillate flowers [4]. The *Acnida* subgenus, which corresponds to the dioecious species, is split into two separate clades in our Mash-based phylogeny (Fig. 2), consistent with the split in previous studies [9, 10]. All dioecious species were placed in one clade, excluding *A. palmeri* and *A. watsonii*, which were placed with monoecious species in the subgenus *Amaranthus*. Although the Dioecious/Pumilus clade in Waselkov et al.’s [10] nuclear phylogeny is congruent with our Mash-based phylogeny, only the sister-species relationships between *A. australis* and *A. cannabinus* and between *A. palmeri* and *A. watsonii* were supported in our analysis. *Amaranthus tuberculatus* was more closely related to *A. floridanus* than to other dioecious species in our study, similar to Stetter and Schmid [9], while *A. arenicola* was more related to *A. greggii*.

The clustering of *A. caudatus*, *A. quitensis*, *A. hybridus*, *A. hypochondriacus*, and *A. cruentus* was consistent with previous tree topologies based on chloroplast markers [10] or biallelic SNPs [9]. We recovered the same relationships among the five monoecious species reported in Xu and Sun’s study [8], which was based on combined AFLP and ISSR datasets. Moreover, the genetic similarity between *A. quitensis* and *A. caudatus* has been suggested to be due to gene flow because the former was often found in *A. caudatus* fields [1, 10].

It is worth mentioning that organellar DNA has been demonstrated not to impact Mash-based phylogeny construction in previous studies, being that their high copy numbers are not represented among low-frequency *k*-mers used in Mash phylogeny [63]. Although assembly- or alignment-free *k*-mer-based methods are optimal in analysis of genome relatedness, they are not without cons in that they are based on assumptions that do not model complex evolutionary processes [19]. A single value is computed as distance per pair of species, and therefore conclusions on the contribution of specific genomic regions to species divergence are difficult to obtain. Moreover, low-depth coverage, variation in library sizes, or missing data could impact the accuracy of MinHash methods whereby distances deviate from true genetic distances [64]. While we did not set out to evaluate these sources of bias, we note that *A. hypochondriacus*, *A. caudatus*, *A. quitensis*, and *B. vulgaris* short reads from the NCBI database had 96, 98, 100, and 124 bp read lengths, respectively, compared to > 130 bp read lengths for other species. Nevertheless, Mash accurately recovered Sauer’s taxonomic ordering of the dioecious amaranths [65] as well as the relationships among monoecious species in the subgenus *Amaranthus*, demonstrating the robustness of Mash in our study.

Also intriguing is the relationship between species clustering from our Mash-based phylogeny and the total TE composition from our dnaPipeTE repeats analysis. *Amaranthus cannabinus* and *A. australis* (60.67% and 60.48%, respectively) had a higher total TE composition than *A. tuberculatus* and *A. floridanus* (56.4% and 54.2%, respectively), followed by *A. acanthochiton*, *A. arenicola*, and *A. greggii*, which were all similar in their total TE composition (51.79%, 53.02%, and 52.84%, respectively) and *A. watsonii* and *A. palmeri*, which had the least TE compositions (44.03% and 37.02%, respectively).

### Whole-sequence alignments and coverage analysis of *Amaranthus palmeri* and *Amaranthus tuberculatus* male-specific regions of the Y

Mapping of Illumina paired-end short reads of sequenced dioecious *Amaranthus* species to draft genomes of both *A. tuberculatus* and *A. palmeri* showed differences in read alignment (Additional file 3: Table S5 – S6). As expected, *A. tuberculatus* reads mapped back to its genome assembly had > 90% reads in proper pairs (Additional file 3: Table S5). Although > 90% of *A. palmeri* reads mapped to its genome assembly, only 77% reads were in proper pairs (Additional file 3: Table S6). Five species, *A. acanthochiton*, *A. arenicola*, *A. australis*, *A. cannabinus*, and *A. floridanus*, had > 70% of paired reads in proper pairs when mapped to *A. tuberculatus* genome while *A. watsonii* had < 67% of paired reads in proper pairs (Additional file 3: Table S5). However, when the short-read sequences were mapped to *A. palmeri* genome, the five species that mapped well to *A. tuberculatus* had < 63% of paired reads in proper pairs, while *A. watsonii* had > 75% of its paired reads in proper pairs (Additional file 3: Table S6). *Amaranthus greggii*, however, had < 66% of its paired reads in proper pairs when mapped to either *A. tuberculatus* or *A. palmeri* draft genomes, perhaps due to its high level of paralogy (discussed above). Structural differences or sequence divergence among the species could have resulted in non-proper pairing of reads for the six genomes when mapped to *A. palmeri* genome. *Amaranthus watsonii*, based on previous phylogenetic studies, including our Mash-based phylogeny, was closely related to *A. palmeri* [10], which is congruent with our mapping results.

**Fig. 3:**
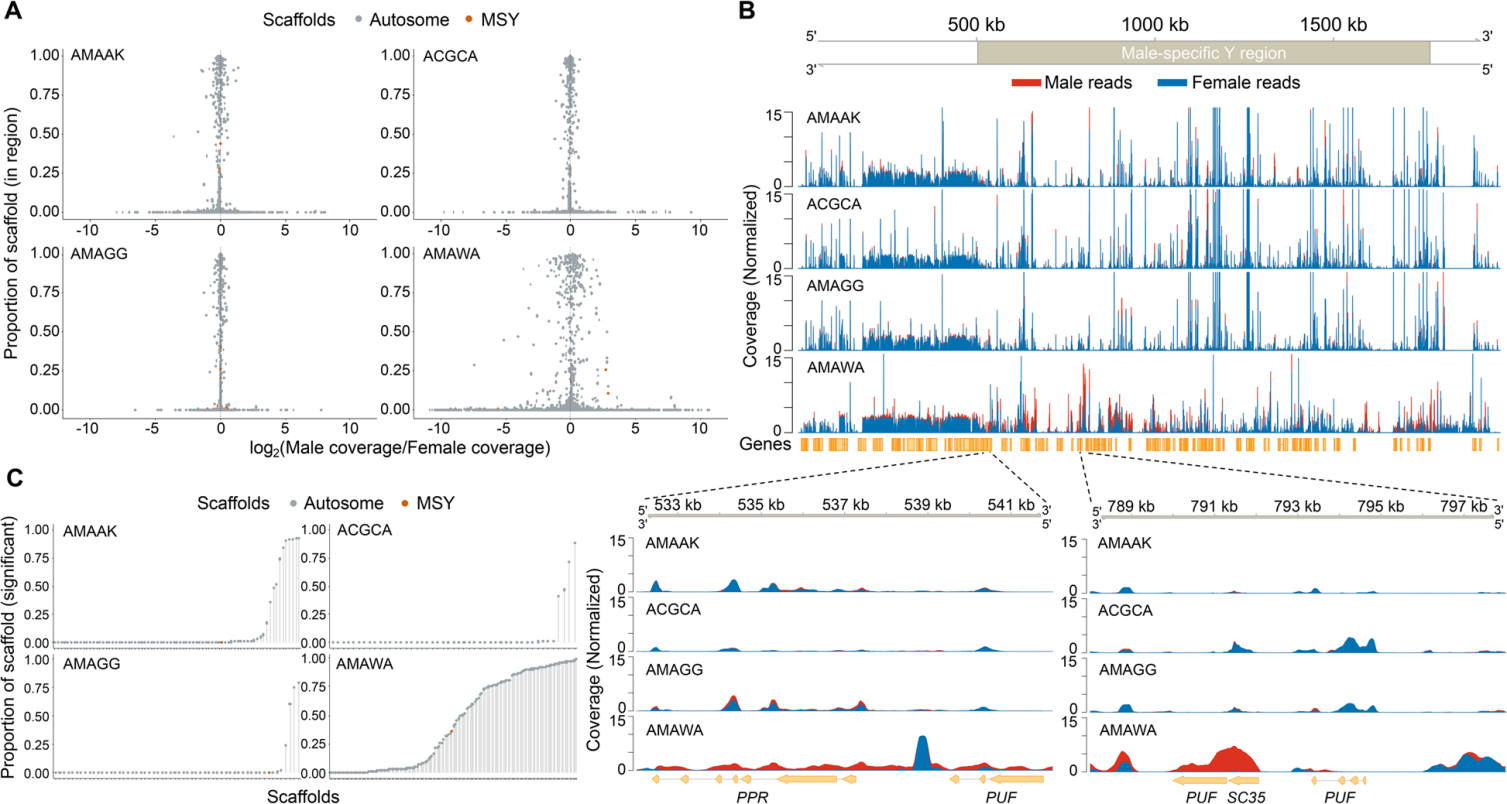
Coverage differences between male and female reads of four dioecious *Amaranthus* species mapped to *A. palmeri* scaffold assembly. **A** Analysis of scaffold regions with male- or female-enriched coverages with DifCover pipeline. The *y*-axis represents the proportion of scaffold the specific region occupies. Orange color is used to indicate regions on the previously identified male-specific region of the Y on scaffold 20. **B** Read alignment coverage from bamCoverage analysis for scaffold 20. Genes exhibiting male-enriched coverages were visualized within a 10-kb window. **C** All significantly different regions for each scaffold plotted as total proportion of the scaffold length. Species name abbreviations represent the EPPO code for the five dioecious species: AMAAK (*Amaranthus acanthochiton*) ACGCA (*Amaranthus cannabinus*), AMAGG (*Amaranthus greggii*), AMAWA (*Amaranthus watsonii*), and AMATU (*Amaranthus tuberculatus*)

Coverage analysis for sequenced reads mapped to the *A. palmeri* genome revealed male- or female-enriched regions across the genome (Fig. 3A, Additional file 5: Table S1 – S4). Only *A. watsonii* mapped reads showed regions with significant spans of male-enriched coverages (Fig. 3A, Additional file 5: Table S4). A total of 84 scaffolds had regions exhibiting male-enriched coverages for *A. watsonii* mapped reads, in which 29 were reported in Neves et al. [23] and 13 were reported in Montgomery et al. [24]. It is worth mentioning that all the male-specific scaffolds reported by Montgomery et al. were among the 42 scaffolds reported by Neves et al. The MSY region of *A. palmeri* was previously identified to span a region of ~ 1.3 Mb on scaffold 20 (503,282–1,770,936 bp), with 121 candidate gene models within the region [23, 24]. Consistent with the two prior studies, scaffold 20 (MSY region) had the highest window and largest bases spanned for male-enriched coverages in our analysis (Fig. 3A, Additional file 5: Table S4). A total of 101 scaffolds had regions with female-enriched coverages, however, several of the scaffolds that were female-enriched were among those exhibiting male-enriched coverages (Additional file 5: Table S4, Additional file 6: Fig. S1). Interestingly, scaffold 19 exhibited significant spans of female-enrichment. Scaffold 19 is 2.23 Mb in length and contains 115 predicted gene models, including pentatricopeptide repeat-containing protein (PPR), serine/arginine-rich splicing factor, and several proteins of unknown function (Additional file 6: Fig. S2). It is worth noting that scaffolds with enrichment more than scaffolds 19 or 20 have shorter lengths (< 200 kb) relative to both scaffolds. Mapped reads of the other three species from both male and female individuals showed no contiguous region was significantly enriched for male or female coverages (Fig. 3A). The fact that some *A. watsonii* female reads also mapped within the MSY region on scaffold 20 suggest that the region is not entirely male-specific, and some portions could be part of the pseudo-autosomal region (PAR) that is still recombining with the X chromosome (Fig. 3B, C).

We identified 11 sex-linked genes with a combined length of 21,680 bp (~ 22 kb) exhibiting male-enriched coverage for *A. watsonii* reads that mapped to the *A. palmeri* MSY region (Additional file 7: Table S1). Only three of these genes had informative annotations, one each as pentatricopeptide repeat-containing protein (PPR), serine/arginine-rich splicing factor, and magnesium protoporphyrin IX methyltransferase. A BLAST search of the remaining 8 genes to the non-redundant protein database on NCBI showed two genes, g4825 and g4829, matched to Zinc finger CCHC-type (*Artemisia annua* L.) and serine/arginine-rich splicing factor (*Arachis hypogea* L.) homologs, respectively, while the remaining 6 genes matched to uncharacterized proteins or had no similarity matches. The *PPR* gene within the sex-determining region was particularly interesting in that six of its seven exons had male-enriched coverages for *A. watsonii* mapped reads, while the three other species had reads from both male and female individuals mapped to the gene (Fig. 3B).

To identify regions within *A. tuberculatus* genome assembly with male- or female-enriched coverages, we included the short reads of female individual of three species, *A. acanthochiton*, *A. cannabinus*, and *A. greggii*, in addition to *A. watsonii*, in that they were farther away from *A. tuberculatus* based on Waselkov et al.’s phylogeny [10]. We reasoned that gene(s) crucial for sex functions should be conserved across species sharing a common dioecy evolutionary event and, therefore, including the most distally related species would identify the most crucial genes. We included previously sequenced short reads of two males and two females of *A. tube*rculatus from Kreiner et al. [66], which were sequenced to 10 × depth.

**Fig. 4:**
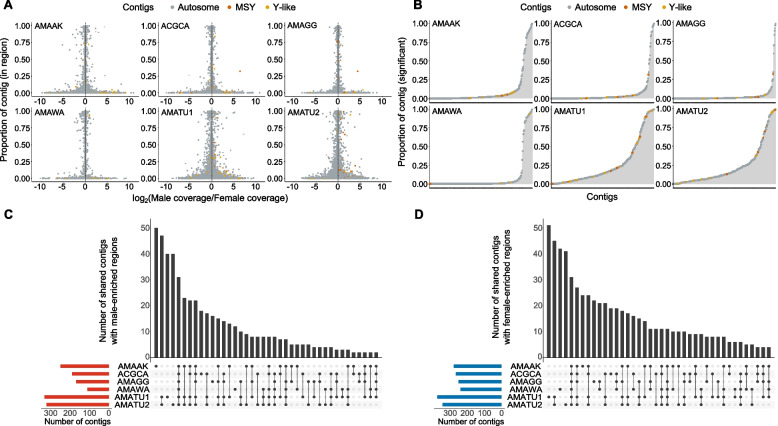
Coverage differences between male and female reads of five dioecious *Amaranthus* species mapped to *A. tuberculatus* contig assembly. **A** Analysis of contig regions with male or female-enriched coverages with DifCover pipeline. The *y*-axis represents the proportion of contig the specific region occupies. Orange color (designated as MSY) is used to indicate regions in the top 10 contigs with both male-specific 15-mer and RAD-tag alignments in Montgomery et al. [24] while yellow color (designated as Y-like) represents regions in 13 other contigs with either the 15-mer or RAD-tag alignments. **B** All significantly different regions for each contig plotted as total proportion of the contig length. **C,D** Upset plots delineating the number of shared contigs with male or female-enriched coverages. Species name abbreviations represent the EPPO code for the five dioecious species: AMAAK (*Amaranthus acanthochiton*) ACGCA (*Amaranthus cannabinus*), AMAGG (*Amaranthus greggii*), AMAWA (*Amaranthus watsonii*), and AMATU (*Amaranthus tuberculatus*)

Among the previously reported MSY contigs, a few were found to exhibit male-enriched coverages for only some species (Fig. 4A). For example, contig 00,001,274 had male-enriched coverages for *A. cannabinus*, *A. greggii*, and *A. tuberculatus*, contig 0,000,298 had male-enriched coverages for *A. greggii* and *A. tuberculatus*, and contig 00,100,752 had male-enriched coverage for *A. cannabinus*, *A. greggii*, and *A. tuberculatus*, although variation existed in the length of bases spanned for the coverages (Additional file 5: Table S5 – S10). Only contig 00,004,323 had male-enriched coverages for all 5 species, while contigs 00,000,336, 00,000,340, 00,003,161, 00,004,353, and 00,100,771 were not enriched for either male- or female-specific coverages for any species. As expected, *A. tuberculatus* had the most significantly enriched contigs (Fig. 4B) and the highest number of contigs (~ 300) for both male- and female-enriched regions, while *A. watsonii* mapped reads had the least number of contigs for male- and female-enriched regions (Fig. 4C). Interestingly, contigs 0,000,298, 00,001,274, 00,001,293, and 00,001,713, which were previously identified as male-specific, had no female-enriched coverages (Additional file 5: Table S5 – S10).

A 200-bp *FLOWERING LOCUS T* (*FT*) on contig 00,000,542 identified as one of the MSY genes in Montgomery et al. [24] was also found to exhibit male-enriched coverages across *A. acanthochiton*, *A. cannabinus*, *A. greggii*, and *A. tuberculatus* while reads from *A. watsonii* did not map to the *FT* gene (Additional file 6: Fig. S3). The gene next to the 200 bp *FT*, although annotated as “unknown,” also showed male-enriched coverage across the three species, including *A. tuberculatus* (Additional file 6: Fig. S3) and had its second and longest exon (14,302–14,525 bp) match to predicted *Beta vulgaris* subsp. *vulgaris Heading date 3a* (LOC104890180) with 84% homology. The 200 bp *FT* also matched to the same *Heading date 3a* locus, but at a different position, and thus we consider this second fragment part of the *FT* gene. In total, there are 4 exons of the *Heading date 3a* in *Beta vulgaris* compared to the two fragments, one with one exon and the other with two exons, in the *A. tuberculatus* contig assembly at this locus.

### Transcription factors and repetitive elements within *Amaranthus palmeri* male-specific region of the Y

Transcription factors (TF) have been implicated in sex functions in flowering plants; however, only a few gene models out of the 121 gene models within the *A. palmeri* MSY region had informative annotation. To therefore identify any transcription factors with potential sex functions among the gene models, a reference plant TF and transcriptional regulator categorization tool, PlantTFcat [67] was used for TF prediction. Seven transcription factors from three family types and four families, one of which was *LBD*, were identified (Additional file 7: Table S2). The TF families with the highest number of genes predicted from the analysis was *CCHC(Zn)* with 4 genes, followed by one gene each for *ASL-LOB*, BED-type(Zn), and GRF (Additional file 7: Table S2). These transcription factors are multifunctional or involved in several processes, including epithelial development, cell adhesion, leaf development, or overall plant growth and development [68–70].

Additional characterization of the ~ 1.3 Mb MSY region of *A. palmeri* for transposable elements revealed consistency with a typical sex determination region, with the accumulation of repetitive sequences and the presence of predominantly male-specific sequences [36, 48, 50]. The MSY region was made up of 78.49% repetitive elements (Additional file 3: Table S4). The long interspersed nuclear elements (LINE/*L1*) made up the highest composition at 19.13%, followed by *copia* and *Ty3* at 15.64% and 12.91%, respectively. The proportion of repeats within the MSY region is higher relative to the entire *A. palmeri* genome (56.03%), indicating that this region has indeed accrued repetitive elements during its evolution. The composition of repeats within this region is also consistent with other studies, e.g., 76.9% of the 1.5 Mb *Mercurialis annua* SDR is made up of repeats, and LTRs were most abundant [71]. Similarly, 77% of the 8.1 Mb *Carica papaya* hermaphroditic specific Y region (HSY) is made up of repeats with *Ty3* being most abundant [72].

## Discussion

We inferred genome characteristics and shallow relationships and gained further understanding of conserved genomic regions with potential roles in sex function among dioecious *Amaranthus* species using comparative genomics. Genome size, repeat proportion, heterozygosity, polyploidy, and GC content are documented genome characteristics that could influence de novo assembly quality [56, 73, 74], and thus genome profiling provides valuable consideration towards a high-quality assembly. *k*-mer analysis of genome sizes for the dioecious *Amaranthus* species were generally consistent with estimates from flow cytometry for previously reported species [9]. Heterozygosity estimates differed across species; although such differences might be species specific, they also could reflect differences due to accessions used, and the number of crosses made to propagate the accessions. Ploidy inference analysis also affirms the previously reported diploid state of the species sequenced in this study [75]. The entire *Amaranthus* genus has been hypothesized to be a paleoallotetraploid [1, 75, 76]; however, *Amaranthus dubius* Mart. Ex Thell. is the only known extant allotetraploid (2*n* = 64) species, with others being diploid (2*n* = 32 or 34). Although, diploidy was inferred for *A. greggii*, the higher number of duplicated sequences or paralogs suggests a possible pre- or post-speciation event could have led to the retention of the sequences.

Repeats analysis revealed transposable elements contributing to genome structure differences in dioecious amaranths. The long terminal repeats (LTRs) proliferation and their elimination is the primary mechanism contributing to genome size variation in dioecious *Amaranthus* species. There is a well-established correlation between genome size and LTR element abundance [77, 78]; however, it is intriguing that the LTR superfamily *copia* element was more abundant than the *Ty3* element for two dioecious species, *A. palmeri* and *A. watsonii*, similar to the pattern for some monoecious *Amaranthus* species. It is possible that the removal of the LTR elements via ectopic recombination differs between the dioecious and monoecious species [78, 79]. The mechanistic process involved in such differential LTR removal however remains elusive. The similar TE pattern between *A. palmeri*-*A. watsonii* and monoecious species is congruent with other studies that have shown some relationships between the two species and the monoecious species [10, 80, 81]. Franssen et al. [82] also suggested that the pollen of *A. palmeri* was less similar to that of the other dioecious *Amaranthus* species sampled (*A. tuberculatus* and *A. arenicola*), and more closely resembled pollen of the monoecious species.

Our complementary repeat discovery methods whereby we analyzed and compared TEs in genome assemblies to TEs from short reads allowed us to identify the abundance of low copy repeats for the amaranths. Various families of transposable elements are known to exist in high copy numbers in the plant genome [83], and repeat discovery tools could identify these high or medium copy repeats [84]. However, it is nontrivial to estimate absolute repeat composition of plant genomes using short-read sequences, and methods relying only on raw reads for genomes with low copy repeats return lower TE contents [60, 85]. Other factors that could result in TE underestimation include short insert size library, novel or diverged repeats in species of interest relative to the annotation database from other species, and difficulty in detecting nested repeats with short reads. Given the analysis of TEs in genome assemblies from our study and the literature, we hypothesize that the composition of TEs in the amaranths ranges from 55 to 75% of the genome. Overall, our findings are congruent with other studies demonstrating the contribution of specific TEs (e.g., LTRs) in genome size variation within a genus, such as in *Oryza* spp. [86].

Interestingly, our phylogenomic analysis of genome relatedness appears to be highly consistent with the early taxonomic works of Jonathan D. Sauer on dioecious amaranths based on comparative morphology and the species’ geographic distributions [2, 3, 33]. For example, *A. arenicola* is closely related to *A. greggii* based on morphology and their proximity around the tropical Gulf coast [3] while *A. watsonii* and *A. palmeri* share an overlapping range, with the former sometimes confused for *A. palmeri* [2]. The sympatry of *A. australis* (southern water hemp) and *A. cannabinus* (eastern water hemp) was also reported, with both species having similar habitat requirements (e.g., salty and fresh water tolerance, and both found in wet sand of coastal marshes) [65]. Of keen interest is the relationship between *A. tuberculatus* and *A. floridanus* in our study, the former being noxious and expands rapidly while the latter is restricted to Florida. The close relationship between both species was also previously established using biallelic SNPs data in SNAPP [9]. *Amaranthus tuberculatus* however has been previously suggested to be more related to *A. arenicola* than many other *Amaranthus* species based on morphology [10, 65]. The higher number of hybrids between *A. tuberculatus *and* A. arenicola* and the limited habitat data for *A. floridanus*, as well as limited to no herbarium collections documenting hybrids between *A. tuberculatus* and *A. floridanus* could have led to the suggestion of their relationships [65]. Mash-based phylogeny have been shown to be robust in species relationship inference with Wascher et al. [63] using it to trace the domestication of cultivated sugar beet to wild relatives in Greece. Similarly, Mash recovered accurate cladograms for polyploid species, where assembly- or alignment-based approaches would have been intractable [64].

Furthermore, our analysis identified regions within the *A. palmeri* genome assembly that are male-enriched, congruent with male-specific scaffolds that were previously reported [23, 24]. We also found scaffold 19 exhibited female-enriched coverages, thus indicating that the scaffold could be part of the X chromosome. Several candidate genes exhibiting male-enriched coverages, including pentatricopeptide repeat-containing protein (PPR) and serine/arginine-rich splicing factor (SC35) were identified within the *A. palmeri* MSY region. Although the genes have no known direct links to sex determination in flowering or dioecious plants, they have been reported to play some roles in sex functions. *PPR*s act as restorers of fertility (*Rf*), i.e., restore partial or normal pollen production to plants via suppression of the cytoplasmic male sterility (CMS) locus [87, 88]. In radish, the *PPR* gene, *Rfo*, was found to restore fertility by specifically downregulating the expression of the CMS locus, *orf138*, in the tapetum of anthers [89]. Whether the *PPR* within *A. palmeri* MSY carries any restoration activity, i.e., has a post-transcriptional action on mitochondrial gene expression, is not known. Recently, a *PPR* was reported as one of the SDR genes in the gymnosperm plant, *Gingko biloba* [90]. Sex-linked genes in other dioecious plant species have been shown to exhibit male-specific coverages within the sex-determining regions, e.g., the sex-determinant factors, *SOFF* in garden asparagus (*Asparagus officinalis* L.) [91], *SyGI* and *FrBy* in kiwifruit (*Actinidia* sp) [40] and *NRT1/PTR6.4* in spinach (*Spinacea oleracea* L.) [45]. For *A. tuberculatus* MSY contigs, we identified several contigs with male-enriched coverages, in which four had no female-enriched coverages for all species. However, only a previously identified *FLOWERING LOCUS T* (*FT*) gene had male-specific coverage for mapped reads of three species, but not for *A. watsonii*, indicating the conservation of the *FT* gene and its possible role in conferring male fitness as previously hypothesized [24]. The difference in mapping pattern between *A. watsonii* and the three other dioecious species is consistent with our hypothesis of a different dioecy evolutionary event in *A. watsonii* and *A. palmeri* relative to the other dioecious amaranths.

### Implications for dioecy evolution within the *Amaranthus* genus

An open question with regard to dioecy within the *Amaranthus* genus has been the evolution of dioecy and the mechanisms involved, and if these could be explained with existing models [36, 92–95]. The phylogenetic study of *Amaranthus* from Waselkov et al. [10] included 58 out of 74 species with all nine dioecious species and is also rooted, providing directionality in ancestry relationship. The Dioecious/Pumilus clade and the Hybridus clade (monoecious) from the study shared a recent common ancestor (Bayesian posterior probability value of 1 and bootstrap support value of 99). Both clades then shared a recent common ancestor with the Galapagos clade, in which all species are monoecious. Although the nuclear-based and the chloroplast-based trees from the study were discordant, characterized by occasional polytomies, less supported nodes, or poorly resolved clades, dioecy within the genus appeared to have originated from a monoecious ancestor.

The *Amaranthu*s genus has been shown to be closely related to *Chamissoa altissima* (Jacq.) Kunth [96], which is hermaphroditic [97]. A maximum-likelihood phylogeny of Amaranthaceae family constructed from 936-nuclear gene supermatrix also showed monophyly of Amaranthoids and Celosioids [15]. The Amaranthoids are characterized by their unisexual flowers while *Celosia argentea*, a member of the Celosioids, has bisexual flowers, indicating the possibility of a hermaphroditic ancestor in the evolution of the *Amaranthus* genus.

It is unclear how dioecy evolved in the genus, whether via hermaphroditism-gynodioecy/androdioecy-dioecy pathway [92], via monoecy-paradioecy-dioecy pathway [93–95, 98], or via an environmentally/physiologically induced mechanism [36, 99, 100]. The origin of dioecy evolution has implications for what mechanisms could be involved in dioecy. Species evolving dioecy via a hermaphroditism-gynodioecy/androdioecy-dioecy pathway have two sex-determinant factors or genes (female suppressor and male activator) that are linked within a region of suppressed recombination on the Y chromosome (MSY or SDR region), which has been observed in *Asparagus officinalis* [41, 91] and *Actinidia* spp [40, 101]. However, species evolving dioecy from hermaphroditism through monoecious populations could utilize a single gene for sex determination, which has been observed in *Diospyros lotus* [39]. The *Amaranthus* genus is made up of 74 species, 9 of which are dioecious while others are monoecious [2, 3, 102, 103], primarily wind-pollinated [28, 76, 104], and no evidence of gynodioecy within the genus points to a likely evolution of dioecy from monoecy. The presence of species with bisexual flowers at the subfamily level (Amaranthoideae) suggests monoecy could have arisen from an ancestral hermaphroditic population, giving rise to a hermaphroditism-monoecy-dioecy pathway [36, 105].

If this is the case, a single gene could thus be sufficient for sex determination in dioecious species of the *Amaranthus* genus. Sex determination in spinach was recently proposed to be controlled by a single gene, *NRT1/PTR6.4* (transporter of nitrate, peptide or hormones), utilizing two pathways for carpel development suppression and stamen initiation [45]. Although the subfamilies Amaranthoideae (*Amaranthus* genus) and Chenopodoideae (*Spinacia* genus) are in the family Amaranthaceae, comprehensive phylogenetic studies have not shown a convincing support for their relationship [15, 106]. A BLAST search of the spinach *NRT1/PTR6.4* against *A. palmeri*, *A. tuberculatus*, or *A. hypochondriacus* on CoGe [107] revealed no orthologs in the amaranths, indicating that *Spinacea* and *Amaranthus* lineage evolved dioecy independently and utilize separate dioecy mechanisms.

Based on our whole-genome analysis of relatedness and other evidence from this study, *A. palmeri* and *A. watsonii* are closely related and likely utilize a similar dioecy mechanism. The other dioecious species form subclades (e.g., close relationship between *Amaranthus tuberculatus* and *A. floridanus*, *A. cannabinus* and *A. australis* and *A. arenicola* and *A. greggii*) within a larger clade. Whether species within this clade and the *A. palmeri*-*A. watsonii* cluster evolved dioecy independently but still recruited the same gene(s) or pathways for such independent evolution is unclear [24, 43]. The availability of chromosome-scale reference genome assemblies and genetic maps for the species will allow further characterization of their sex chromosomes.

## Conclusions

We report genome characteristics, including size, heterozygosity, and ploidy for seven newly sequenced dioecious species within the *Amaranthus* genus. Although our transposable element analysis does not capture the full suite of repetitive elements in the respective genomes, it offered a new view of TE dynamics among the dioecious *Amaranthus* species, especially for the species with no high-quality reference or even draft genomes. Furthermore, a pattern of TE proliferation is emerging in the genus, in which some dioecious species have a higher proportion of *Ty3* than *copia* elements, but the reverse is the case for *A. palmeri*, *A. watsonii*, and some monoecious species. It is unclear what the “correct” topology for dioecious species relationship is within the *Amaranthus* genus. Nevertheless, we provide additional evidence supporting early taxonomic relationships among the dioecious *Amaranthus* species based on comparative morphology, i.e., close relationship between *A. palmeri* and *A. watsonii*, *A. australis* and *A. cannabinus*, and *A. tuberculatus* and *A. floridanus*, as well as their relationship to the monoecious species in the subgenus *Amaranthus*. We report 11 gene models, including a pentatricopeptide repeat-containing protein and serine/arginine-rich splicing factor within the *A. palmeri* MSY region that also exhibit male-specific coverages for *A. watsonii*. In addition, a previously reported *FT* within an *A. tuberculatus* MSY contig was found to exhibit male-specific coverage for three species but not for *A. watsonii*. Overall, our findings support the previous hypothesis that dioecy evolved separately in *A. tuberculatus* and *A. palmeri*.

## Methods

### Plant material, DNA extraction, and Illumina sequencing

Accessions of seven dioecious amaranths were obtained from USDA Germplasm Resources Information Network (GRIN) (Additional file 1: Table S1). Voucher specimens of all accessions sequenced in this study can be found at the Illinois Natural History Survey (ILLS) Herbarium at the University of Illinois Robert A. Evers Laboratory. Voucher barcodes are included in Additional file 1: Table S1. Seeds were grown in containers filled with a growing media that included Sunshine LC1 (Sun Gro Horticulture, 770 Silver Street Agawam, MA) growing mix, soil, peat, and torpedo sand (3:1:1:1 by weight). Two or three young leaves were harvested from each species following flower formation and visual identification of gender. Leaf tissues collected were frozen in liquid nitrogen and stored in − 80 °C pending DNA extraction. Genomic DNA was extracted from one male of each species and from one female each of *A. acanthochiton*, *A. cannabinus*, *A. greggii*, and *A. watsonii* following standard CTAB protocol [108]. DNA integrity was determined using a spectrophotometer (Nanodrop1000 Spectrophotometer, Thermo Fisher Scientific, 81 Wyman Street, Waltham, MA 02,451) and by resolving the DNA on 1% agarose gel by electrophoresis. The absence of band shearing or smearing indicated high molecular weight DNA with sufficient purity and quality required for sequencing. The eleven DNA samples were submitted to the Roy J. Carver Biotechnology Center at the University of Illinois, Urbana–Champaign for sequencing. Shotgun genomic libraries were prepared with Hyper Library construction kit from Kapa Biosystems (Roche, Basel, Switzerland), and the libraries were size selected, pooled, quantitated by qPCR, and paired-end sequenced (2 × 150 bp) on one S4 lane for 151 cycles on Illumina NovaSeq6000. Sequences of seventeen other species belonging to either the *Amaranthus* genus or broadly a member of the family Amaranthaceae were downloaded from the NCBI database. Sequencing platforms for these genomes varied from Illumina Hiseq 2500 to Novaseq 6000 (Additional file 1: Table S1).

### Genome size, heterozygosity, and ploidy analysis

The genome sizes for the species sequenced were estimated with GenomeScope v2.0 [Ranallo-Benavidez et al. [51]; https://github.com/tbenavi1/genomescope2.0]. A *k*-mer length, *k*, of 21 was chosen for genome size estimation based on the recommendations from the authors, which was seen as a balance between speed of computation and accuracy. *K*-mer frequencies were generated from the adapter trimmed Illumina sequences for each of the nine dioecious amaranth species with Jellyfish v2.3.0 [109] using parameters: count -C -m 21 -s 3G -t 6 /dev/fd/0 -o output_reads.jf, and histograms of *k*-mer frequencies were obtained using the “histo” sub-command and –high = 1,000,000 flag. Genome sizes were then estimated from the histograms using GenomeScope v2.0 with parameters: -i reads.histo -o output_dir -k 21 -m 1,000,000. The *k*-mer histograms obtained from previous steps were further analyzed with two *k*-mer-based tools, CovEST v0.5.6 [ [53]; https://github.com/mhozza/covest] and FindGSE [ [54]; https://github.com/schneebergerlab/findGSE]. We used both the “basic” and “repeats” model of CovEST with default parameters, except -r 150. The “basic” model is for simple genomes without repeats; however, species of the *Amaranthus* genus have been shown to be made of at least 50% repetitive elements [52, 58]. Moreover, the “repeats” model is error-aware, accounts for repeat structures, and performs well on data with low sequencing coverage [53].

The ploidy levels for each of the genomes were also estimated using Smudgeplot v0.2.3 [ [51]; https://github.com/KamilSJaron/smudgeplot]. *K*-mer frequencies were first generated using KMC v3.1.1 [ [110]; https://github.com/tbenavi1/KMC] with parameters: -k21 -t10 -m30 -ci1 -cs10000 @FILES kmer_counts tmp and then converted to *k*-mer frequency histogram using parameters: kmc_tools transform kmcdb histogram species_k21.hist -cx10000. “FILES” contain the raw read names for forward and reverse reads. The “smudgeplot.py cutoff species_k21.hist L/U” was then used to estimate *k*-mer coverage thresholds from the histogram file. *K*-mers in the coverage range from L to U were extracted with the command “kmc_tools transform,” and smudge_pairs command was used to reduce the file to compute set of *k*-mer pairs. The smudgeplots showing proposed ploidy for each of the genomes were then generated with coverages of identified *k*-mer pairs (i.e., species_coverages.tsv file) using “smudgeplot.py plot” command. Haploid *k*-mer coverages were estimated directly from the histogram generated by KMC, rather than supplied from GenomeScope output.

### Transposable element analysis of unassembled *Amaranthus* genomes

We analyzed repetitive elements in the unassembled Illumina raw reads from males of sequenced dioecious *Amaranthus* species using a similarity-based clustering tool, RepeatExplorer2 on a dedicated cloud galaxy instance [57, 111] (https://repeatexplorer-elixir.cerit-sc.cz/galaxy). The sex of *A. palmeri* was unidentified by the authors in their study [112]; however, we included the raw reads sequence for comparison. A recommendation from authors of RepeatExplorer2 is that coverage greater than 1 × be avoided while coverage between 0.1 and 0.5 × is optimal. We therefore subsampled all reads to 0.3 × with rasusa v0.6.1 [113] (https://github.com/mbhall88/rasusa) using parameters: -i r1.fq -i r2.fq –coverage 0.3 –genome-size estimated-genomesize-from-genomescope -o out.r1.fq -o out.r2.fq -s 15. For each species: 1,263,202 (*A. acanthochiton*), 1,142,178 (*A. arenicola*), 1,739,786 (*A. australis*), 1,424,616 (*A. cannabinus*), 1,212,116 (*A. floridanus*), 1,237,964 (*A. tuberculatus*), 1,394,368 (*A. greggii*), 806,748 (*A. watsonii*), and 910,966 (*A. palmeri*) read pairs were kept after subsampling. Reads of *A. hybridus* (894,080), *A. hypochondriacus(*1,279,884), and *A. cruentus* (979,906) subsampled to 0.3 × were also included for comparisons. The FastQ read pairs for each species were quality filtered and interleaved with “Preprocessing of FASTQ paired-end reads” tool in RepeatExplorer Utilities on the galaxy instance. The interleaved reads were then analyzed for repeats with RepeatExplorer2 clustering tool using default parameters. The clusters of repeats within each supercluster were manually inspected to ensure accuracy of the automated repeat prediction. Repeat proportions from the curated cluster table were then estimated using the “Repeat proportions from CLUSTER_TABLE” tool also on the galaxy instance.

We complemented our repeat discovery approach using dnaPipeTE v1.3.1 [61]. First, we constructed a representative repeat library for the amaranths from the genome of *A. hypochondriacus* [58]. De novo identification of species-specific repeats in the genome was carried out with RepeatModeler v2.0.2 using default parameters [114]. A curated RepBase database (RepeatmaskerEdition-20181026) [115] was combined with RepeatMasker default Dfam3.2 database, and “famdby.py” utility was used to query the combined database to obtain a library of “viridiplantae” repeats with parameters: -i RepeatMaskerLib.h5 families –format fasta_name –include-class-in-name –ancestors –descendants “viridiplantae.” We performed additional LTR structural analysis using LTR_retriever pipeline [116], first by analyzing the genome with LTR_harvest [117] from genometools v1.6.0 using the parameters: -minlenltr 100 -maxlenltr 7000 -mintsd 4 -maxtsd 6 -motif TGCA -motifmis 1 -similar 85 -vic 10 -seed 20 -seqids yes, and then through LTR_FINDER_parallel [118] using default parameters. Output from both LTR_harvest and LTR_FINDER_parallel were concatenated and analyzed with LTR_retriever v2.9.0 to obtain a non-redundant LTR library using default parameters [116]. The non-redundant LTR library was then merged with “viridiplantae” repeats and the species-specific consensus library of repeats. To reduce redundancy, the final repeat library was clustered using CD-HIT-EST v4.6 [119] with parameters: -c 0.8 -G 1 -s 0.9 -aL 0.8 -aS 0.8 -M 5000 -T 6 -i. The repeat library was then used with dnaPipeTE for repeat discovery in each species using parameters: -RM_lib repeat library -genome_size estimated-genome-size-from-genomescope -genome_coverage 0.3 and other parameters default. Prior to repeat analysis with dnaPipeTE, we first mapped the raw reads of each species to *A. hypochondriacus* chloroplast genome (GenBank accession number KX279888) [120], and subsequently to *Beta vulgaris* mitochondrial genome (GenBank accession number BA000009) [121] using Bowtie v2.4.4 [122] while keeping only non-aligned reads with parameters: -p 32 -X 1000 –un-conc. This step was taken to avoid the assembly of organellar DNA into contigs that could spuriously be annotated as repeats.

For TE quantification in available *Amaranthus* genome assemblies, we constructed species-specific libraries for each of the species following the method described previously. Final curated libraries were then used to analyze and annotate repeats in the genomes using RepeatMasker v4.1.2-p1 (http://www.repeatmasker.org/RepeatMasker/) with default parameters.

### Mash-based whole-genome phylogenetic analysis

Quality of the Illumina raw reads obtained from 17 species in the NCBI database was accessed with FastQC (https://www.bioinformatics.babraham.ac.uk/projects/fastqc/) and aggregated with MultiQC v1.5 [123]. Low-quality bases and adapters were then removed with Trimmomatic [124] using parameters: ILLUMINACLIP:TruSeq3-PE.fa:2:30:10:2:True LEADING:3 TRAILING:3 MINLEN:36. *Chenopodium quinoa* raw reads (Project number PRJNA821252) had Nextera adapter sequences and were thus removed using parameters: ILLUMINACLIP:NexteraPE-PE.fa:2:30:10:2:True LEADING:3 TRAILING:3 MINLEN:36. To then determine relatedness among the seven sequenced dioecious *Amaranthus* genomes in this study and the 17 other species from the public repository, we used an assembly/alignment-free tool, Mashtree v1.2.0 with the following parameters: –mindepth 0 –numcpus 6 *FORWARD.fastq.gz > mashtree.dnd [62]. Mashtree handles only single reads; therefore, we used only forward reads from the paired read sequences. We included the female reads of four species (*A. acanthochiton*, *A. cannabinus*, *A. greggii*, and *A. watsonii*) to ascertain the robustness of the alignment-free approach in that males are expected to cluster with the respective females of the species. Mashtree uses a *k*-mer strategy in a two-step approach, first adopting the MinHash algorithm of Mash to create genome sketches [19], and second using the sketches to determine distances between genomes as a pairwise distance matrix, which is subsequently used to build a neighbor-joining tree in QuickTree [125]. The output tree (.dnd) from Mashtree was visualized and annotated with FigTree v1.4.4 (https://github.com/rambaut/figtree).

### Whole-sequence alignments and coverage analysis of *Amaranthus palmeri* and *Amaranthus tuberculatus* male-specific region of the Y

Demultiplexing of Fastq raw reads was carried out with Illumina bcl2fastq v2.20 Conversion Software, and quality control, including adapter trimming from the reads, was carried out by the sequencing facility. A total of ~ 6.23 Gb of raw reads were obtained corresponding to 528,703,130 (*A. acanthochiton* female, 127 × genome coverage), 604,304,170 (*A. acanthochiton* male, 145 ×), 533,480,886 (*A. arenicola* male, 142 ×), 642,410,494 (*A. australis* male, 121 ×), 676,006,832 (*A. cannabinus* female, 144 ×), 592,414,420 (*A. cannabinus* male, 126 ×), 572,691,874 (*A. floridanus* male, 143 ×), 540,070,720 (*A. greggii* female, 118 ×), 525,935,576 (*A. greggii* male, 115 ×), 489,955,162 (*A. watsonii* female, 183 ×), and 525,324,158 (*A. watsonii* male, 197 ×) read pairs. The quality of reads for *A. palmeri* and *A. tuberculatus* obtained from the NCBI database was accessed as previously described.

All reads (seven males and four females) were then mapped to the *A. palmeri* and *A. tuberculatus* draft genomes [47] with BWA-MEM v0.7.5 using default settings [126]. The tool “fixmate” within SAMtools v1.14 was used to fill mate coordinates and insert size fields [127], and duplicates in the read alignments were marked with Picard v2.26.9 (http://broadinstitute.github.io/picard/). SAMtools flagstat was then used to compute overall summary statistics of read alignment. Alignment files for each species were filtered with SAMtools to remove reads with mapping quality (MAPQ) < 5, alternative hits (tag XA:Z), and supplementary alignments (tag SA:Z). Coverage analysis was then carried out with the filtered alignments using DifCover [128, 129], which puts into consideration the modal coverage of male and female samples for depth normalization and also accounts for problematic region such as highly repetitive regions or gaps. DifCover was recently implemented in a computational workflow (SexFindR) that identifies sex chromosomal regions [130]. The estimated genome-wide coverages represented as the ratio of log2 male-to-female reads mapped to both *A. palmeri* and *A. tuberculatus* genome assemblies were then plotted with the R packages tidyverse [131] and ggpubr (https://github.com/kassambara/ggpubr).

Additionally, read coverages for scaffold 20, the location of the *A. palmeri* MSY region, were calculated and normalized from the filtered alignments using bamCoverage v3.5.1 [132] with parameters: -b input.bam -o output_cov -of bigwig -bs 20 -r region-of-interest –effectiveGenomeSize estimated-genome-size-from-genomescope –normalizeUsing RPGC –smoothLength 60 –extendReads 150 –ignoreDuplicates –exactScaling -p 5. Coverages and gene annotations were then plotted and visualized using rtracklayer v1.54.0 [133], GenomicFeatures v1.46.5 [134], and Gviz v1.38.3 [135] in R v4.1.2 [136].

We also accessed the presence of *NRT1/PTR6.4* recently proposed as a sex determinant in spinach [45] in the genomes of *A. hypochondriacus*, *A. palmeri*, and *A. tuberculatus* genomes using a BLAST search on CoGE [107]. Although both spinach (subfamily Chenopodoideae) and amaranth (subfamily Amaranthoideae) lineages are paraphyletic from previous phylogeny [15, 106], our hypothesis that the gene could be present in the amaranths was informed by the two lineages belonging to the family Amaranthaceae and show some relationships in the previous trees.

### Transcription factors and repetitive elements within *Amaranthus palmeri* male-specific region of the Y

Identification of transcription factors among candidate gene models within the *A. palmeri* MSY region was carried out using PlantTFcat [67]. A custom repeat library for *A. palmeri* genome [47] was also prepared as previously described for the *A. hypochondriacus* genome. The library of repeats was then used to analyze and annotate repetitive elements within the MSY region of *A. palmeri* using RepeatMasker v4.1.2-p1 with default parameters.

## Supplementary Information

**Additional file 1:** **Table S1.** TableS1 – *Amaranthus* species and other members of Caryophyllales used in this study.

**Additional file 2: Figures S1 – S12.** Fig S1 – Genome size estimate and ploidy level inference for *A. acanthochiton*. Fig S2 – genome size estimate and ploidy level inference for *A. arenicola*. Fig S3 – Genome size estimate and ploidy level inference for *A. australis*. Fig S4 – Genome size estimate and ploidy level inference for *A. cannabinus*. Fig S5 – Genome size estimate and ploidy level inference for *A. floridanus*. Fig S6 – Genome size estimate and ploidy level inference for *A. tuberculatus*. Fig S7 – Genome size estimate and ploidy level inference for *A. greggii*. Fig S8 – Genome size estimate and ploidy level inference for *A. watsonii*. Fig S9 – Genome size estimate and ploidy level inference for *A. palmeri*. Fig S10 – Genome size estimate and ploidy level inference for *A. hybridus*. Fig S11 – Genome size estimate and ploidy level inference for *A. hypochondriacus*. Fig S12 – Genome size estimate and ploidy level inference for *A. cruentus*.

**Additional file 3: Tables S1 – S6.** Table S1 – Genome size estimates (bp) from CovEST basic and repeats model, and findGSE. Table S2A – Composition (%) of repeats identified in *Amaranthus* species using RepeatExplorer2. Table S2B – Composition (%) of repeats identified in *Amaranthus* species using dnaPipeTE. Table S3 – Repeat composition for *A. hypochondriacus*, *A. hybridus* and *A. tuberculatus* genome assemblies. Table S4 – Composition of repeats for *A. palmeri* genome assembly and the male-specific region of the Y. Table S5 – Statistics of short-reads alignment to *A. tuberculatus* male contig assembly. Table S6 – Statistics of short-reads alignment to *A. palmeri* male scaffold assembly.

**Additional file 4: Figures S1 – S12.** Fig S1 – Proportion of repeats in subsampled *A. acanthochiton* genome. Fig S2 – Proportion of repeats in subsampled *A. arenicola* genome. Fig S3 – Proportion of repeats in subsampled *A. australis* genome. Fig S4 – Proportion of repeats in subsampled *A. cannabinus* genome. Fig S5 – Proportion of repeats in subsampled *A. floridanus* genome. Fig S6 – Proportion of repeats in subsampled *A. tuberculatus* genome. Fig S7 – Proportion of repeats in subsampled *A. greggii* genome. Fig S8 – Proportion of repeats in subsampled *A. watsonii* genome. Fig S9 – Proportion of repeats in subsampled *A. palmeri* genome. Fig S10 – Proportion of repeats in subsampled *A. hybridus* genome. Fig S11 –Proportion of repeats in subsampled *A. hypochondriacus* genome. Fig S12 – Proportion of repeats in subsampled *A. cruentus* genome.

**Additional file 5: Tables S1 – S10.** Table S1 – Coverage analysis for *A. acanthochiton* male and female reads mapped to *A. palmeri* genome. Table S2 – Coverage analysis for *A. cannabinus* male and female reads mapped to *A. palmeri* genome. Table S3 – Coverage analysis for *A. greggii* male and female reads mapped to *A. palmeri *genome. Table S4 – Coverage analysis for *A. watsonii* male and female reads mapped to *A. palmeri* genome. Table S5 – Coverage analysis for *A. acanthochiton* male and female reads mapped to *A. tuberculatus* genome. Table S6 – Coverage analysis for *A. cannabinus* male and female reads mapped to *A. tuberculatus* genome. Table S7 – Coverage analysis for *A. greggii* male and female reads mapped to *A. tuberculatus* genome. Table S8 – Coverage analysis for *A. watsonii* male and female reads mapped to *A. tuberculatus* genome. Table S9 – Coverage analysis for *A. tuberculatus* male (ERR3220246) and female (ERR3220227) reads mapped to *A. tuberculatus* genome. Table S10 – Coverage analysis for *A. tuberculatus* male (ERR3220310) and female (ERR3220231) reads mapped to *A. tuberculatus* genome.

**Additional file 6: Figures S1 – S3.** Fig S1 – Upset plot of shared scaffolds with male- or female-enriched coverages. Fig S2 - Reads alignment coverage of male-to-female individuals for four dioecious *Amaranthus* species across scaffold 19. Fig S3 – Reads alignment coverage of male-to-female individuals for five dioecious *Amaranthus* species across *FLOWERING LOCUS T* (*FT*) on contig 00000542.

**Additional file 7: Tables S1 – S2.** Table S1 – Gene models within *A. palmeri* male-specific region of the Y exhibiting male-enriched coverage for *A. watsonii* mapped reads. Table S2 – Transcription factors identified within *A. palmeri* male-specific region of the Y.

## Data Availability

Raw reads data generated or analyzed in this study are available through the National Center for Biotechnology Information (NCBI) under project number PRJNA836903 [137]. Additional datasets, including cluster table of repeats from RepeatExplorer2, Mash distance matrix, Mashtree tree (.dnd), and R scripts used in the analyses are available on figshare 10.6084/m9.figshare.19735501[138].

## Abbreviations

AFLP: Amplified fragment length polymorphism
CoGe: Comparative genomics platform
DNA: Deoxyribonucleic acid
EPPO: European and Mediterranean Plant Protection Organization
G3PDH: Glycerol-3-phosphate dehydrogenase
GRF: Growth-regulating factors
ISSR: Inter simple sequence repeats
ITS: Internal transcribed spacer
LINE: Long interspersed nuclear element
LTR: Long terminal repeat
MSC: Multispecies coalescent
MSY: Male-specific Y
PAR: Pseudo-autosomal region
RAD: Restriction site associated DNA
SDR: Sex-determining region
SNAPP: SNP and AFLP Package for Phylogenetic analysis
SNPs: Single-nucleotide polymorphisms
TE: Transposable elements
TF: Transcription factors
