# Supplementary material for: Genomic profiling of dioecious Amaranthus species provides novel insights into species relatedness and sex genes
Source: BMC Biol. 2023 Feb 20;21:37. doi: 10.1186/s12915-023-01539-9 (PMC9940365; doi:10.1186/s12915-023-01539-9)
Supplement: Supplementary file 2 — Additional file 2: Figures S1 – S12. Fig S1 – Genome size estimate and ploidy level inference for A. acanthochiton. Fig S2 – genome size estimate and ploidy level inference for A. arenicola. Fig S3 – Genome size estimate and ploidy level inference for A. australis. Fig S4 – Genome size estimate and ploidy level inference for A. cannabinus. Fig S5 – Genome size estimate and ploidy level inference for A. floridanus. Fig S6 – Genome size estimate and ploidy level inference for A. tuberculatus. Fig S7 – Genome size estimate and ploidy level inference for A. greggii. Fig S8 – Genome size estimate and ploidy level inference for A. watsonii. Fig S9 – Genome size estimate and ploidy level inference for A. palmeri. Fig S10 – Genome size estimate and ploidy level inference for A. hybridus. Fig S11 – Genome size estimate and ploidy level inference for A. hypochondriacus. Fig S12 – Genome size estimate and ploidy level inference for A. cruentus. [file 12915_2023_1539_MOESM2_ESM.docx]

**Additional file 2**


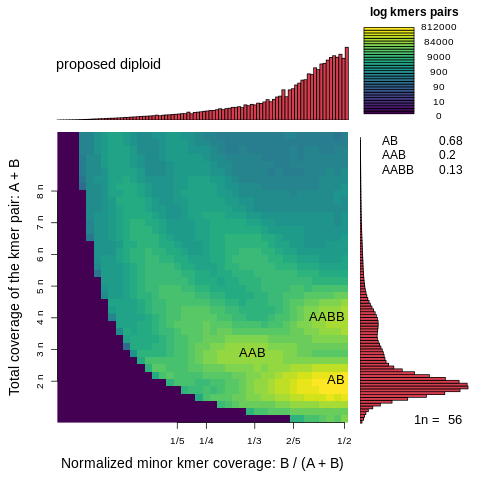
*
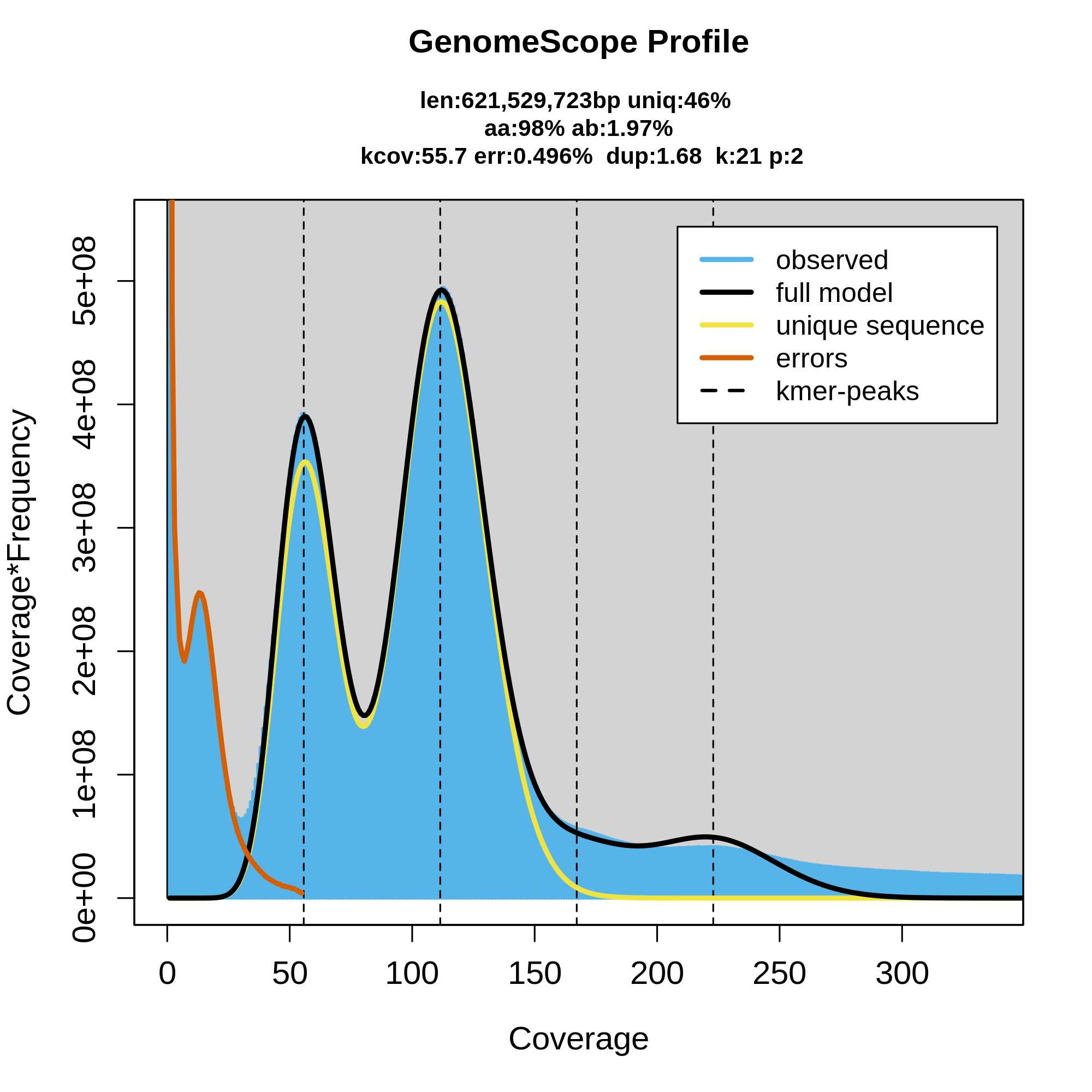
*

**Figure S1.** Genome size estimate and ploidy level inference for *A. acanthochiton*


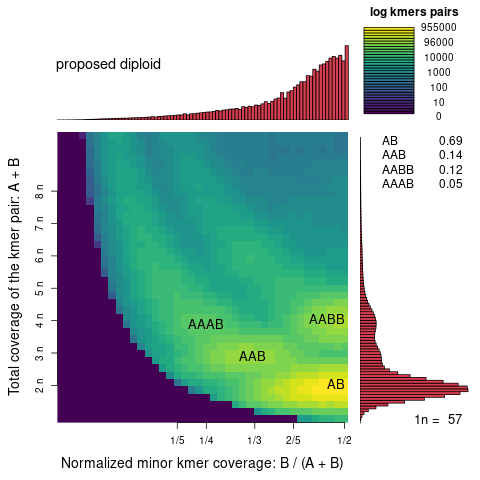
*
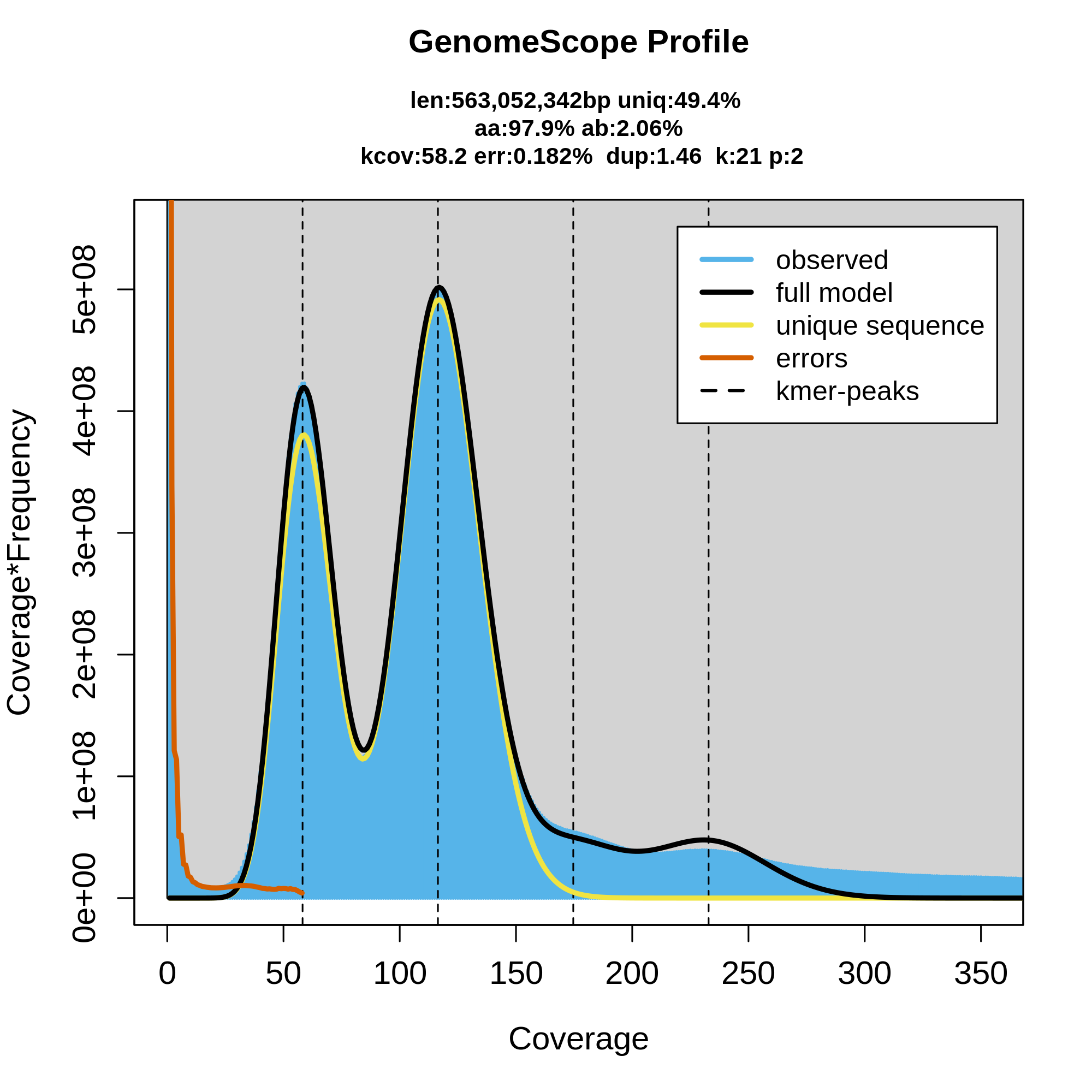
*

**Figure S2.** Genome size estimate and ploidy level inference for *A. arenicola*


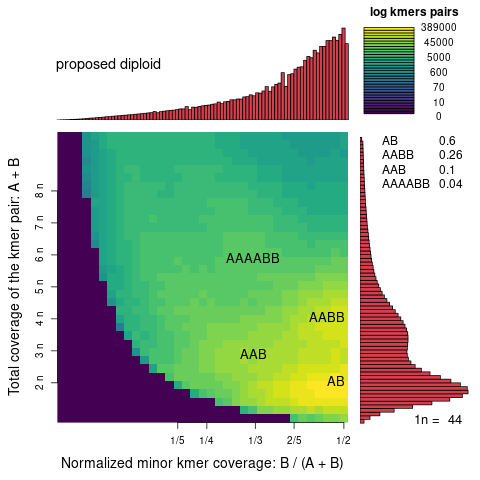
*
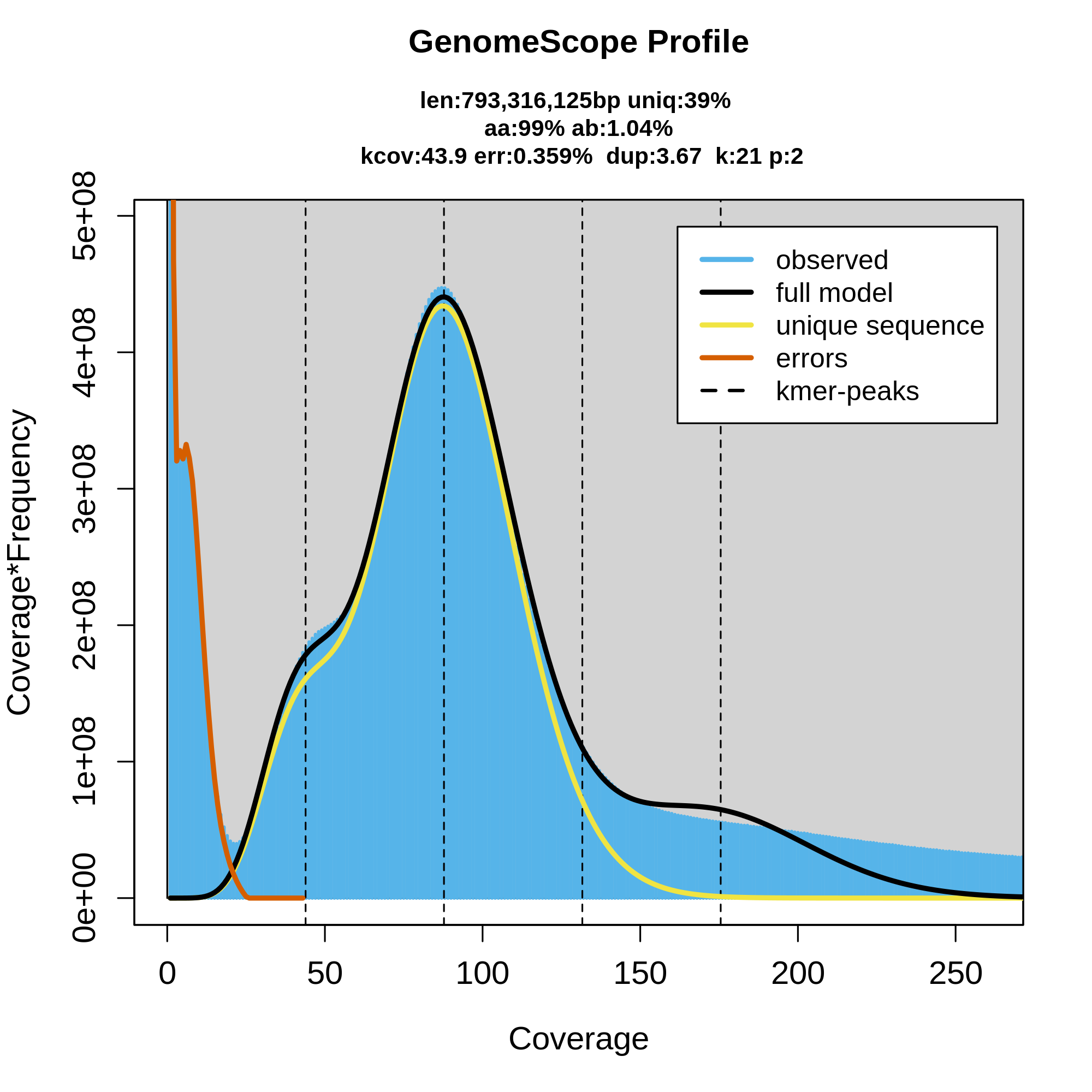
*

**Figure S3.** Genome size estimate and ploidy level inference for *A. australis*


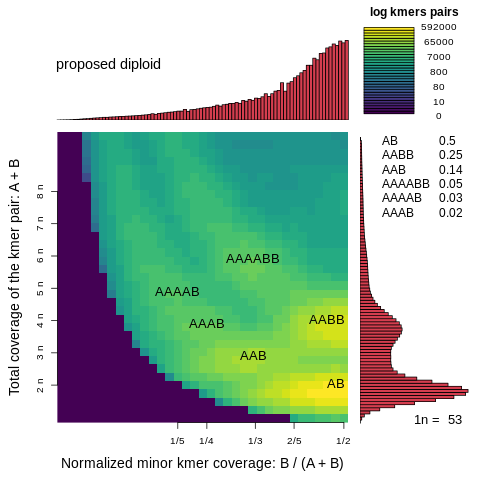
*
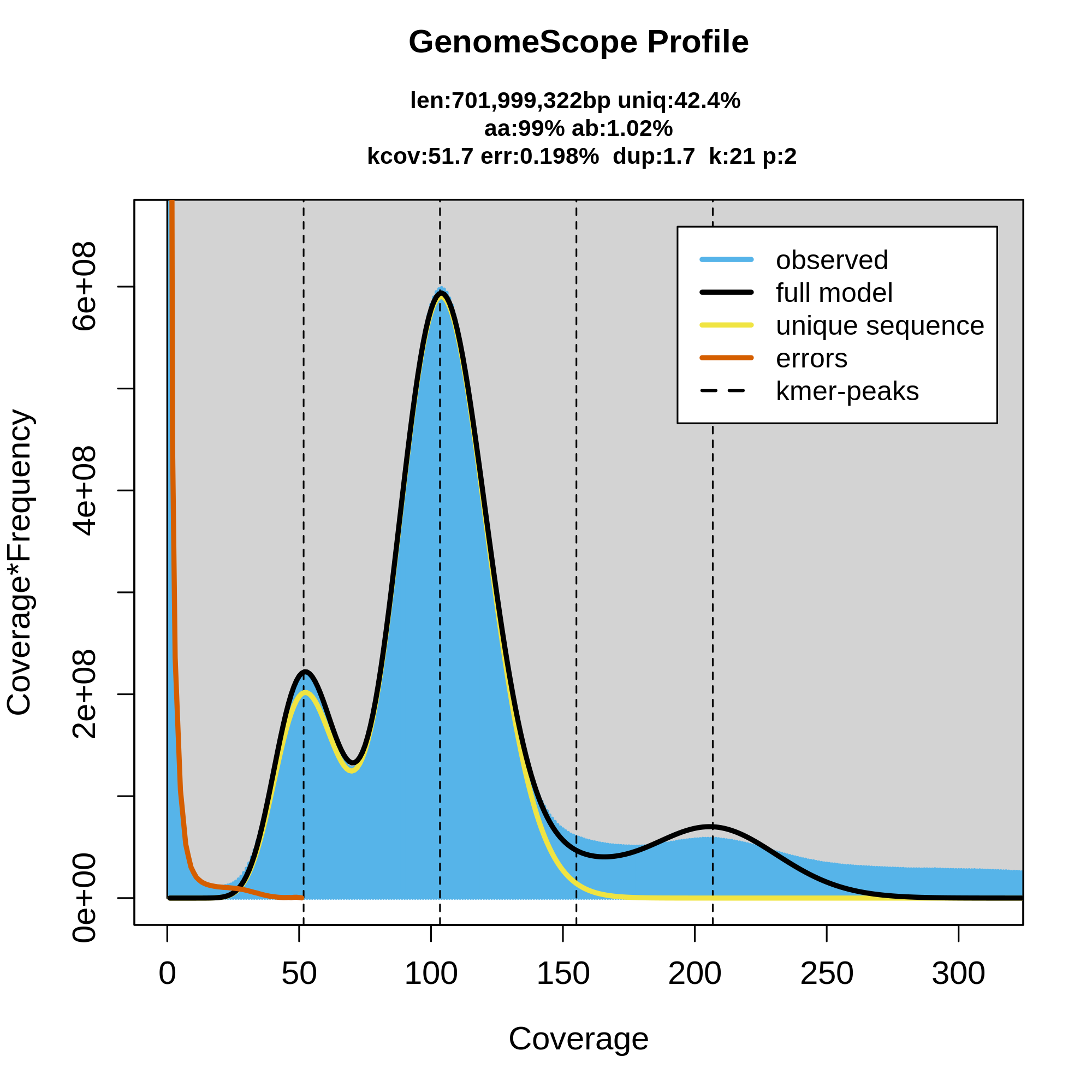
*

**Figure S4.** Genome size estimate and ploidy level inference for *A. cannabinus*


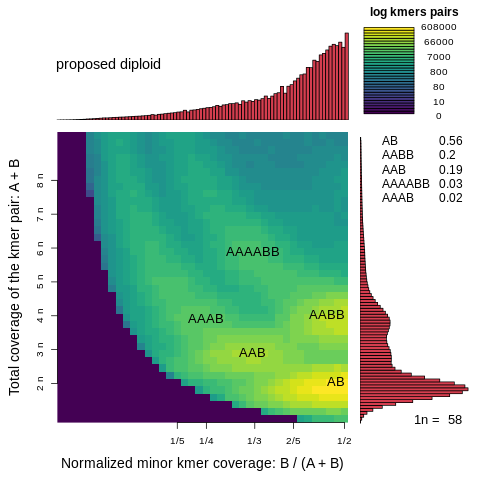

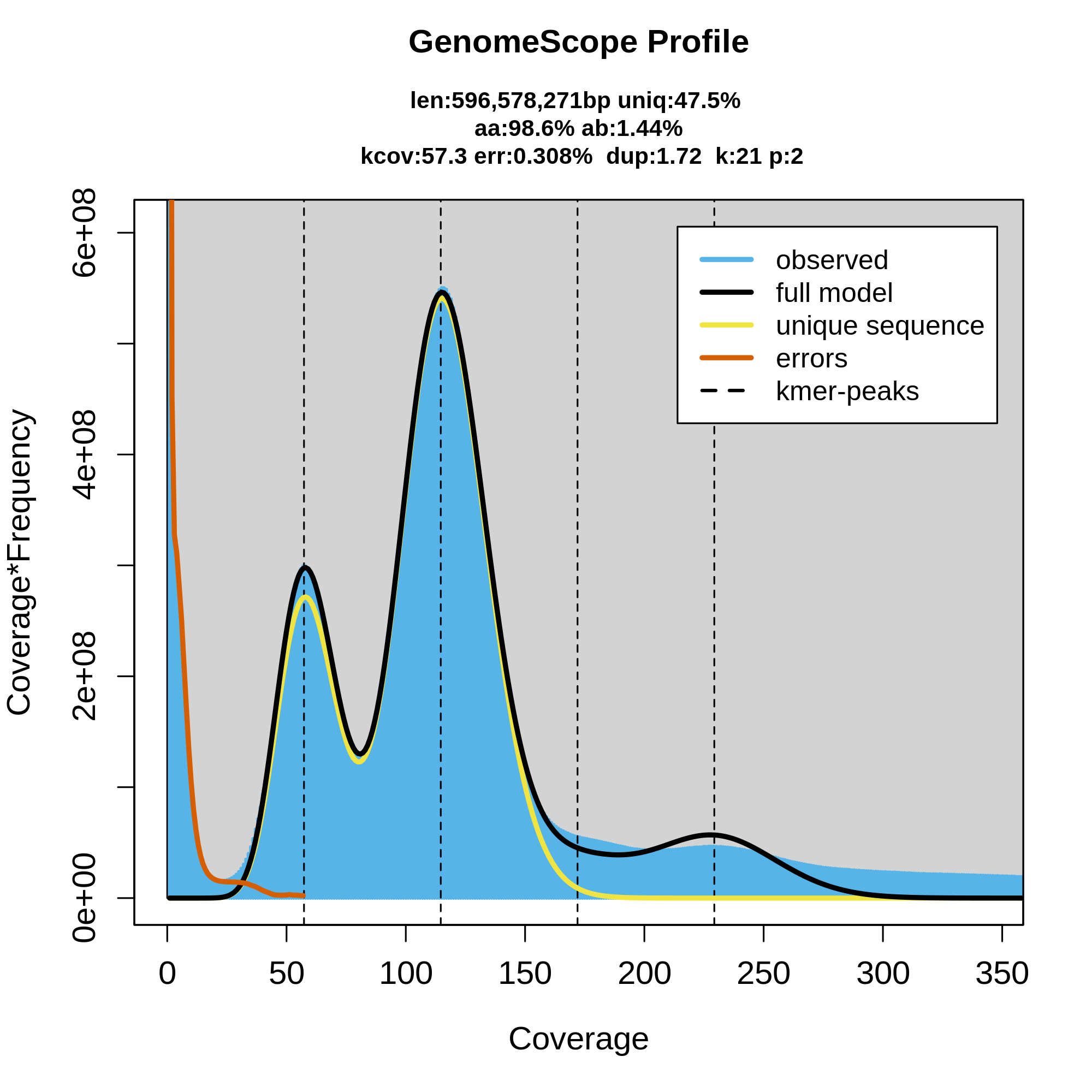


**Figure S5.** Genome size estimate and ploidy level inference for *A. floridanus*


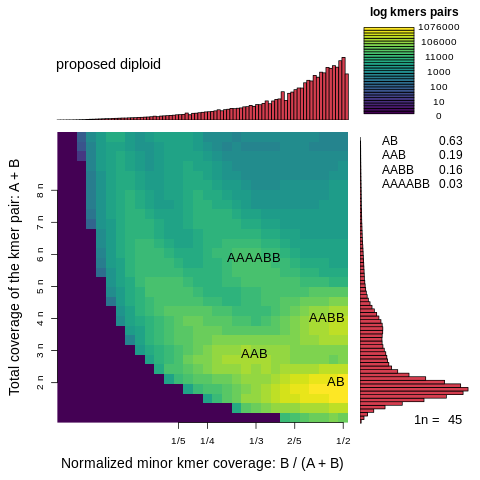


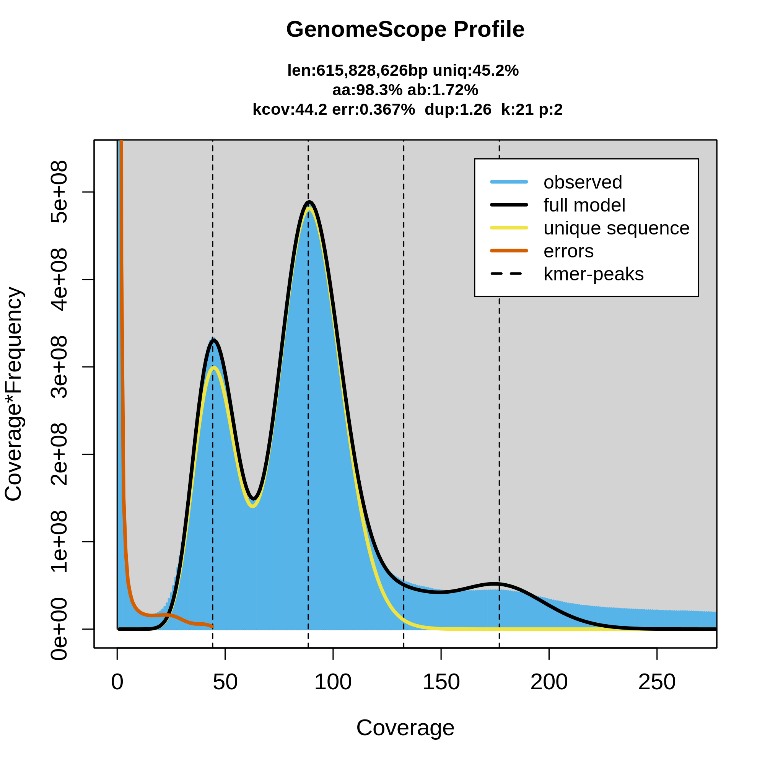


**Figure S6.** Genome size estimate and ploidy level inference for *A. tuberculatus*


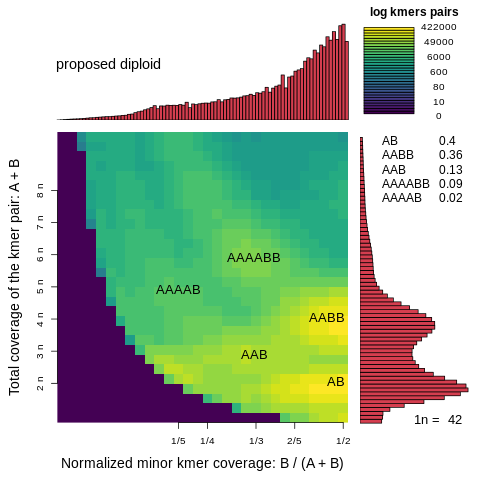
*
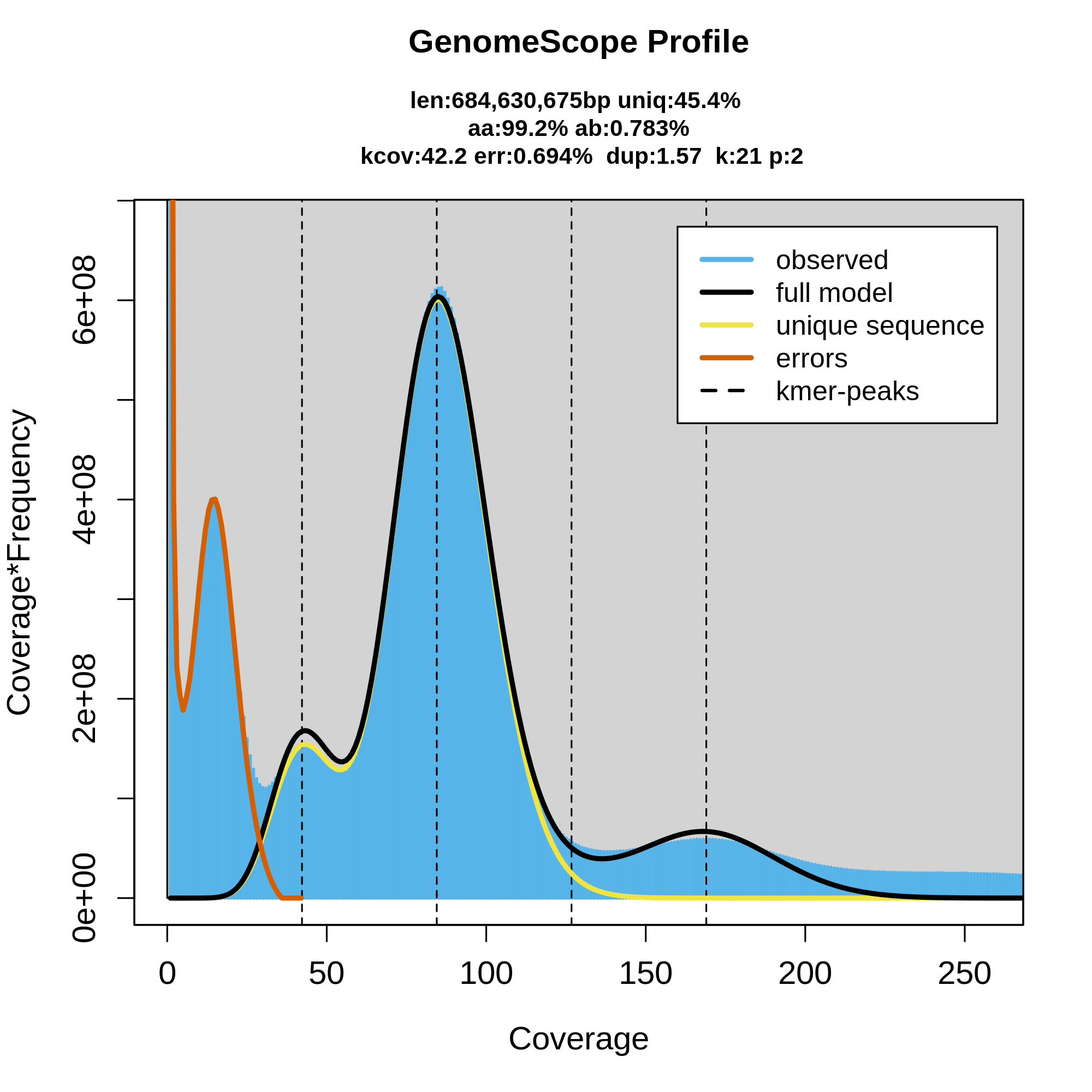
*

**Figure S7.** Genome size estimate and ploidy level inference for *A. greggii*


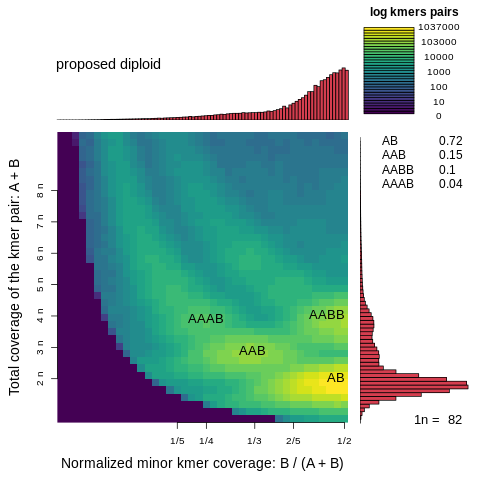


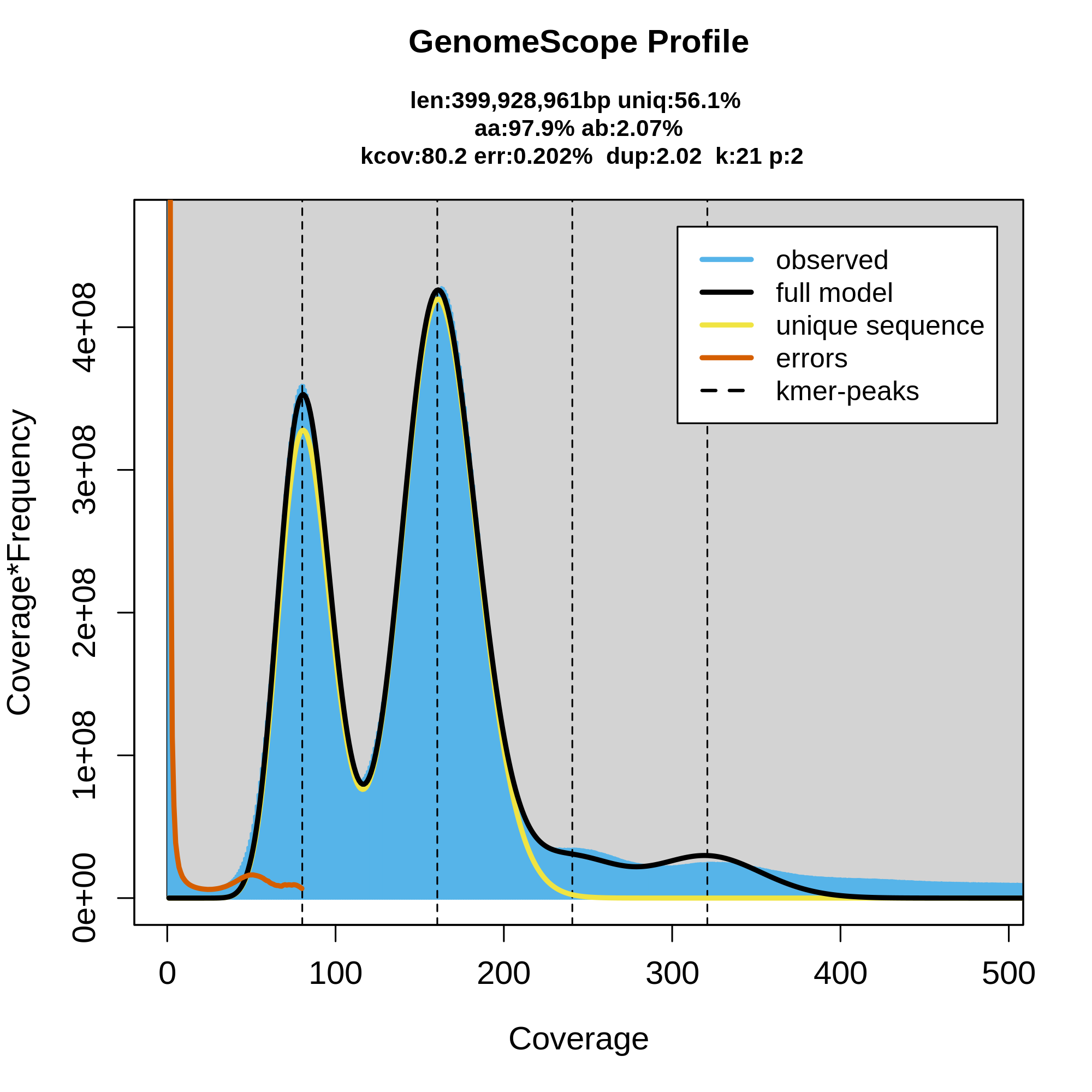


**Figure S8.** Genome size estimate and ploidy level inference for *A. watsonii*


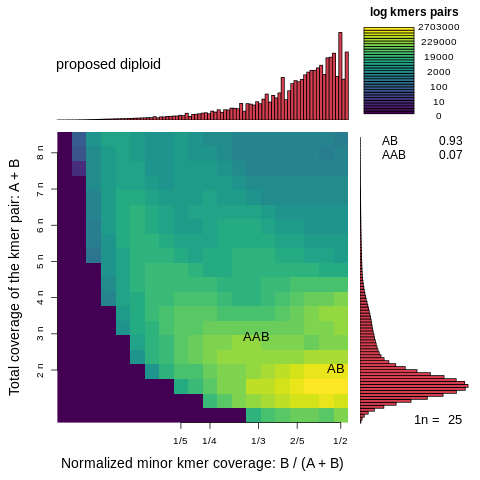

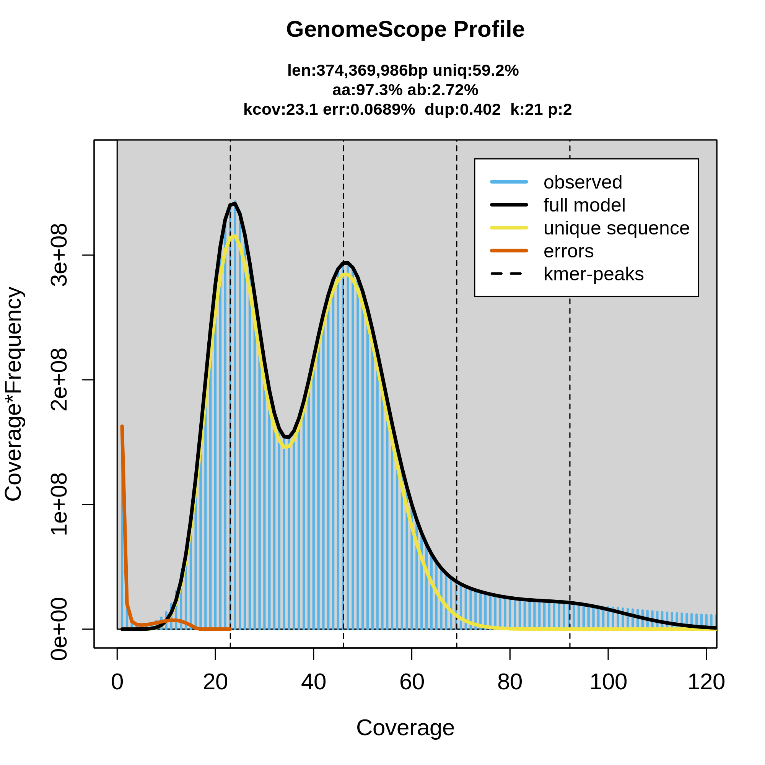


**Figure S9.** Genome size estimate and ploidy level inference for *A. palmeri*


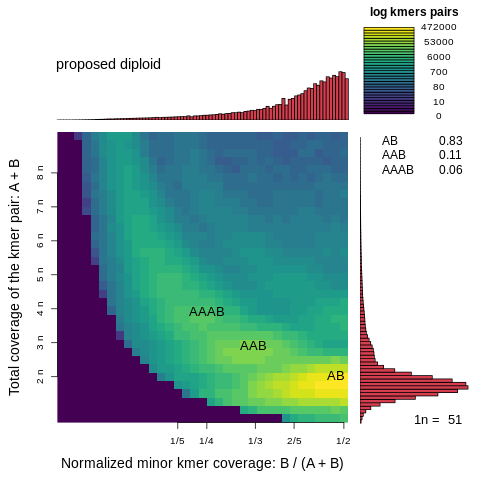


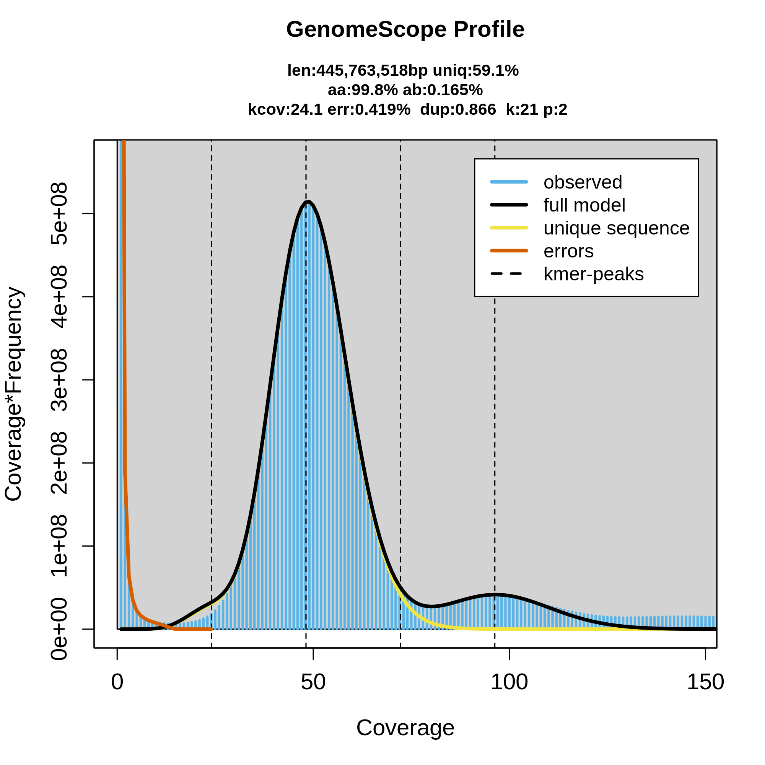


**Figure S10.** Genome size estimate and ploidy level inference for *A. hybridus*


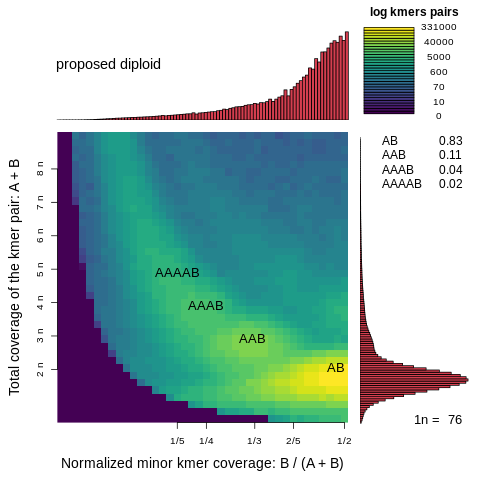

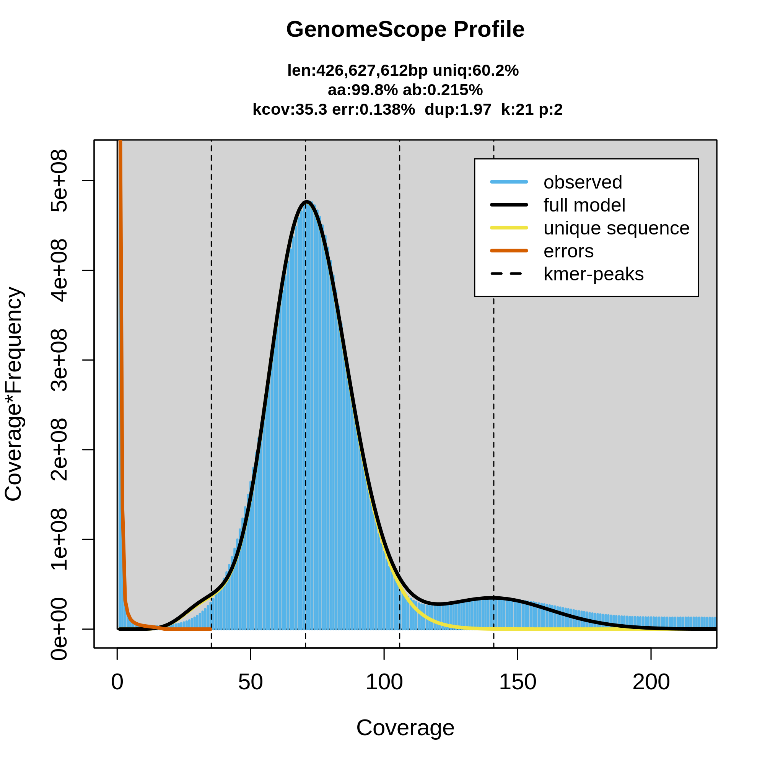


**
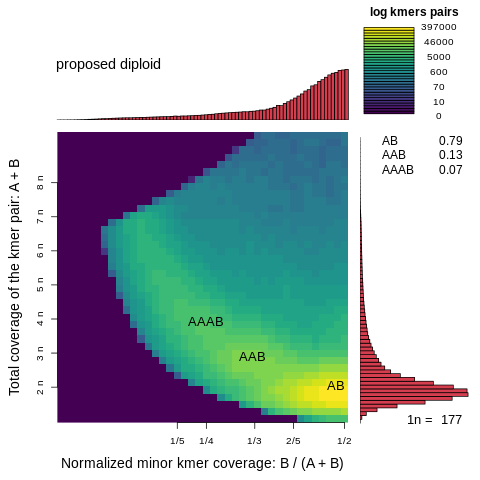
Figure S11.** Genome size estimate and ploidy level inference for *A. hypochondriacus*


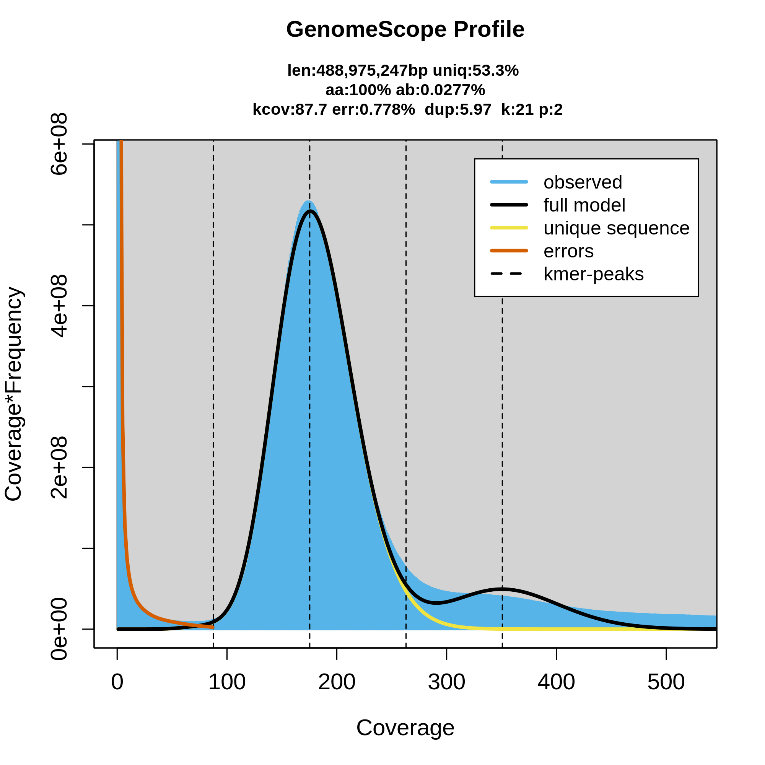


**Figure S12.** Genome size estimate and ploidy level inference for *A. cruentus*
