# Supplementary material for: Genomic profiling of dioecious Amaranthus species provides novel insights into species relatedness and sex genes
Source: BMC Biol. 2023 Feb 20;21:37. doi: 10.1186/s12915-023-01539-9 (PMC9940365; doi:10.1186/s12915-023-01539-9)
Supplement: Supplementary file 4 — Additional file 4: Figures S1 – S12. Fig S1 – Proportion of repeats in subsampled A. acanthochiton genome. Fig S2 – Proportion of repeats in subsampled A. arenicola genome. Fig S3 – Proportion of repeats in subsampled A. australis genome. Fig S4 – Proportion of repeats in subsampled A. cannabinus genome. Fig S5 – Proportion of repeats in subsampled A. floridanus genome. Fig S6 – Proportion of repeats in subsampled A. tuberculatus genome. Fig S7 – Proportion of repeats in subsampled A. greggii genome. Fig S8 – Proportion of repeats in subsampled A. watsonii genome. Fig S9 – Proportion of repeats in subsampled A. palmeri genome. Fig S10 – Proportion of repeats in subsampled A. hybridus genome. Fig S11 –Proportion of repeats in subsampled A. hypochondriacus genome. Fig S12 – Proportion of repeats in subsampled A. cruentus genome. [file 12915_2023_1539_MOESM4_ESM.docx]

**Additional file 4**


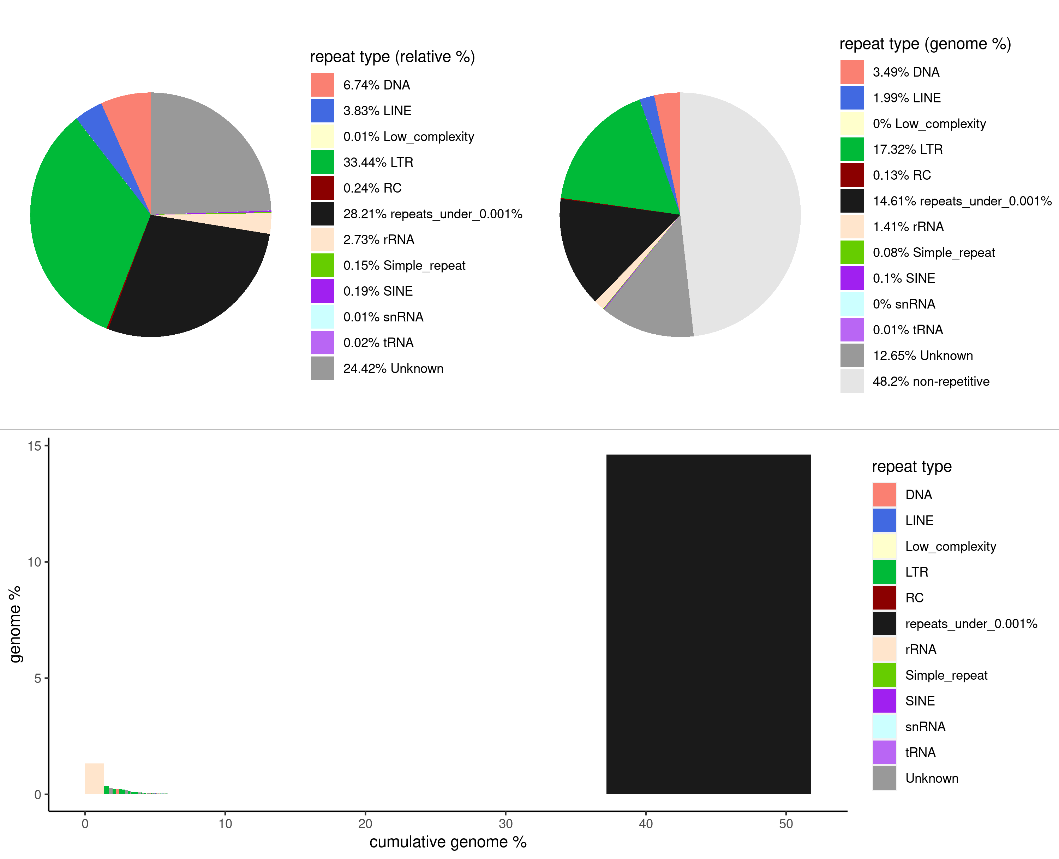


**Figure S1.** Proportion of repeats in subsampled *A. acanthochiton* genome


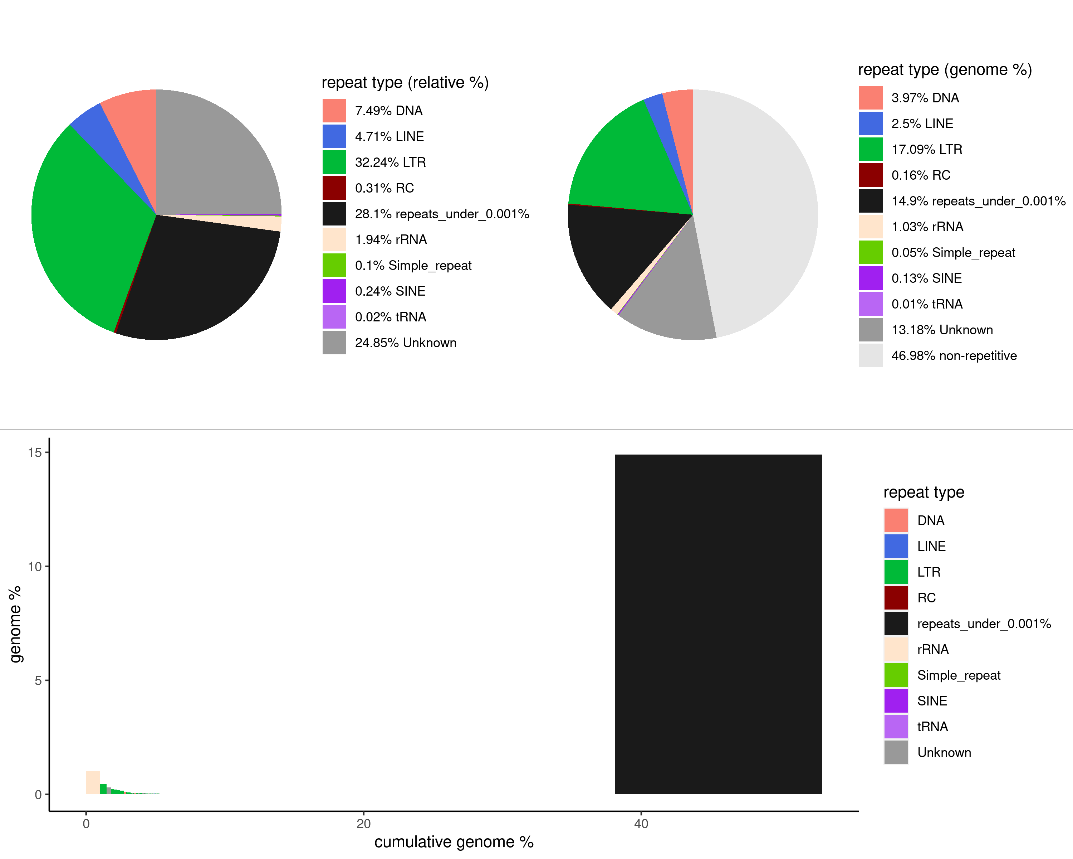


**Figure S2.** Proportion of repeats in subsampled *A. arenicola* genome


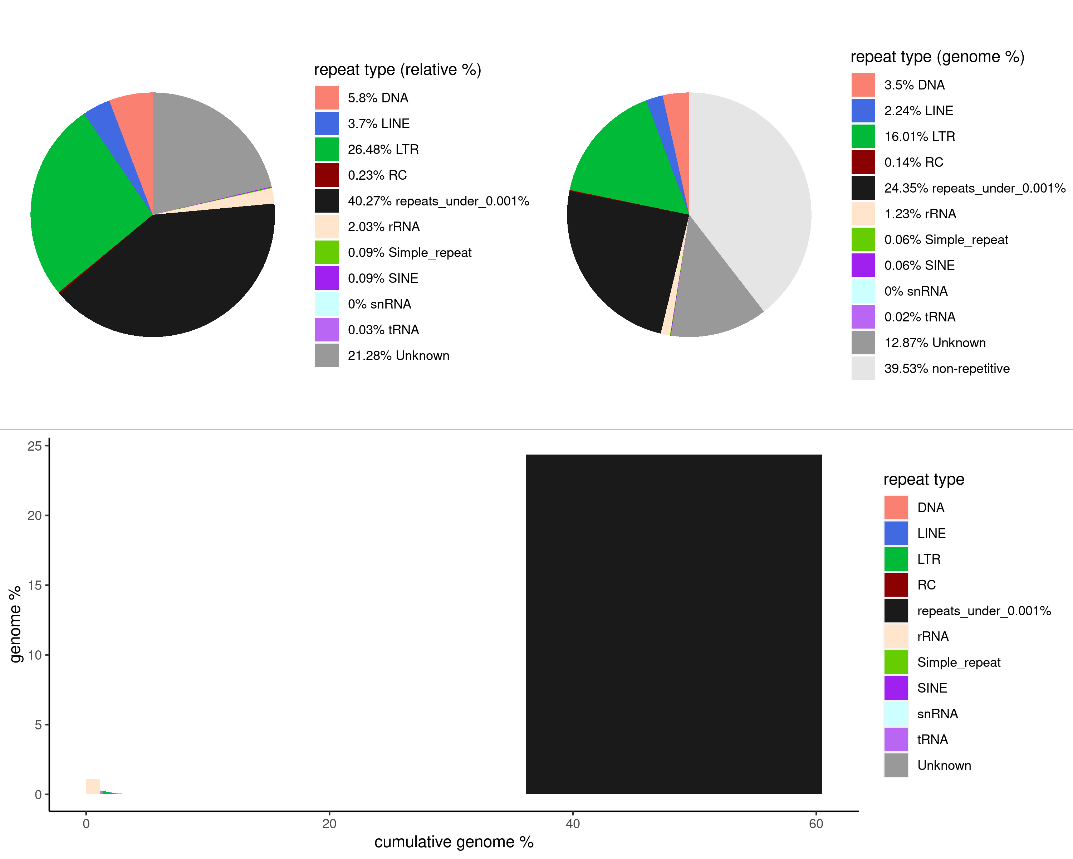


**Figure S3.** Proportion of repeats in subsampled *A. australis* genome


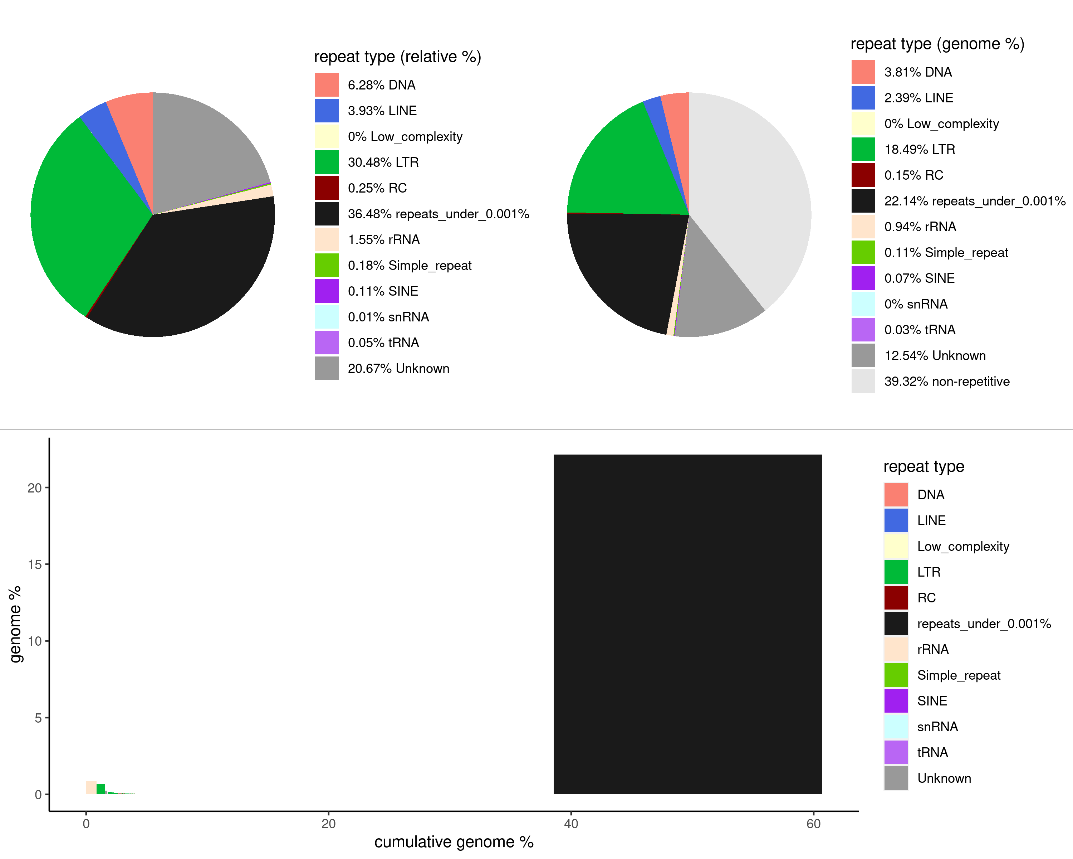


**Figure S4.** Proportion of repeats in subsampled *A. cannabinus* genome


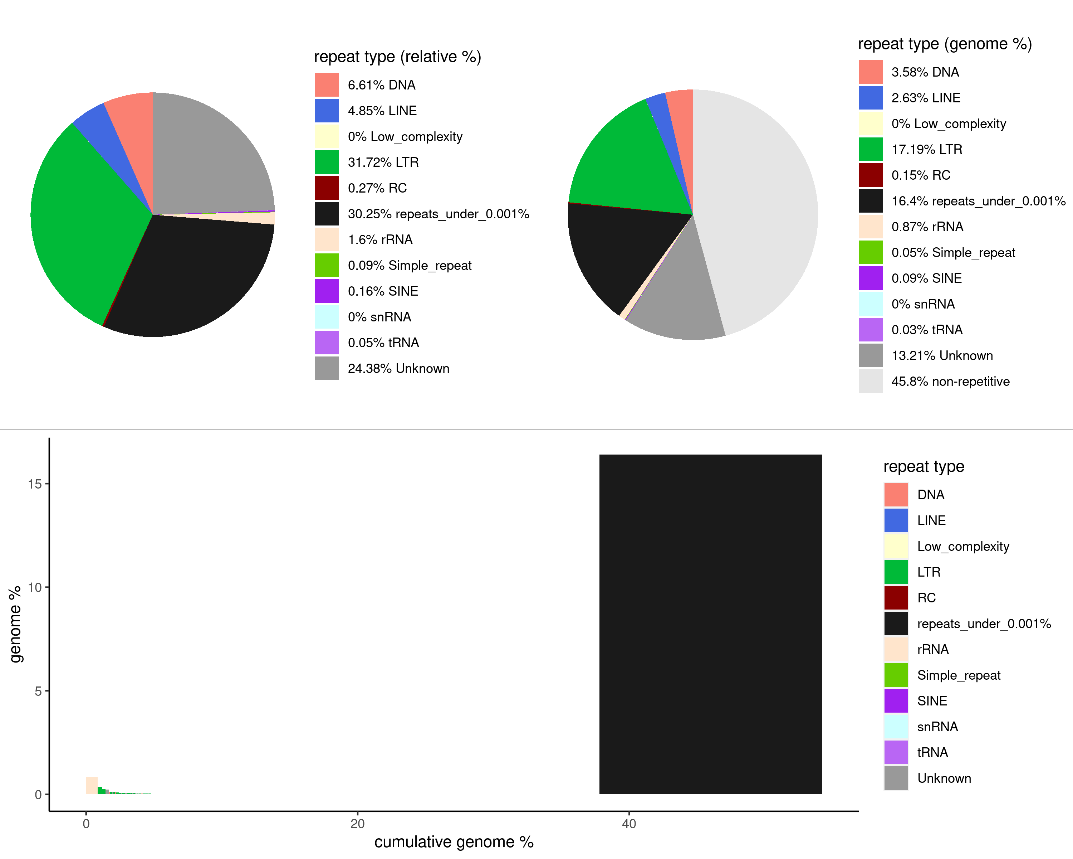


**Figure S5.** Proportion of repeats in subsampled *A. floridanus* genome


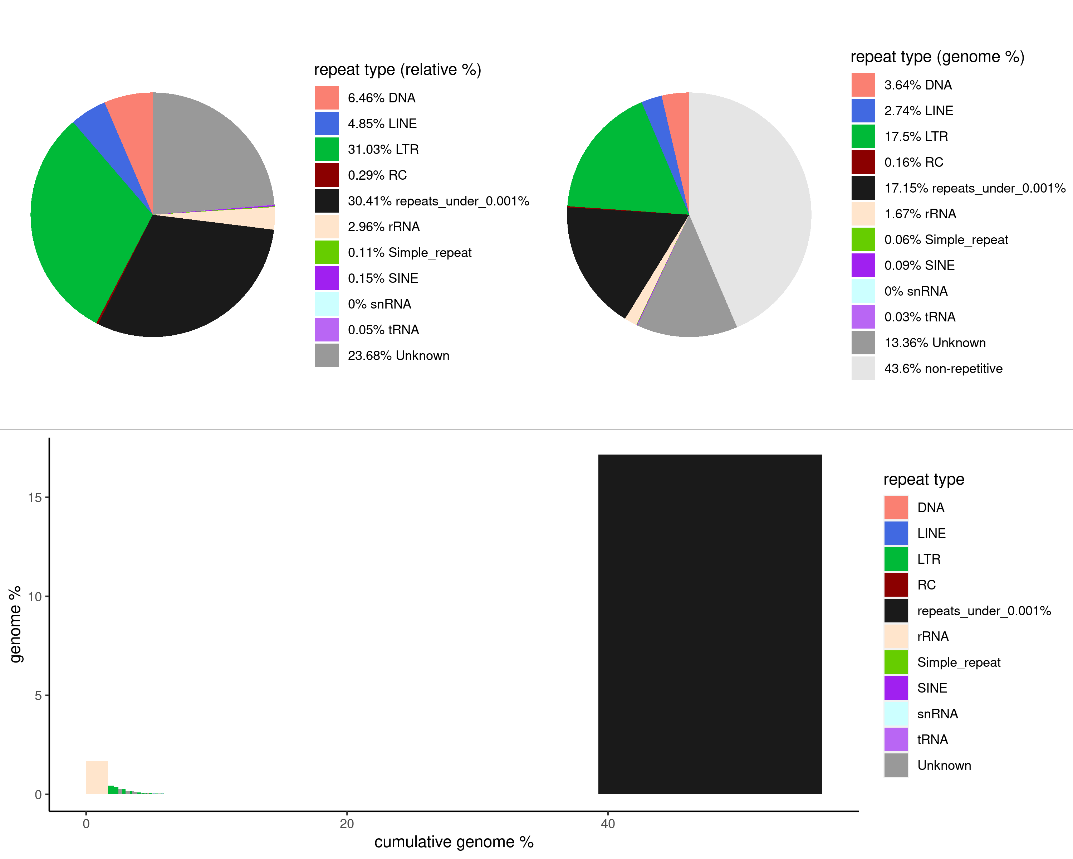


**Figure S6.** Proportion of repeats in subsampled *A. tuberculatus* genome


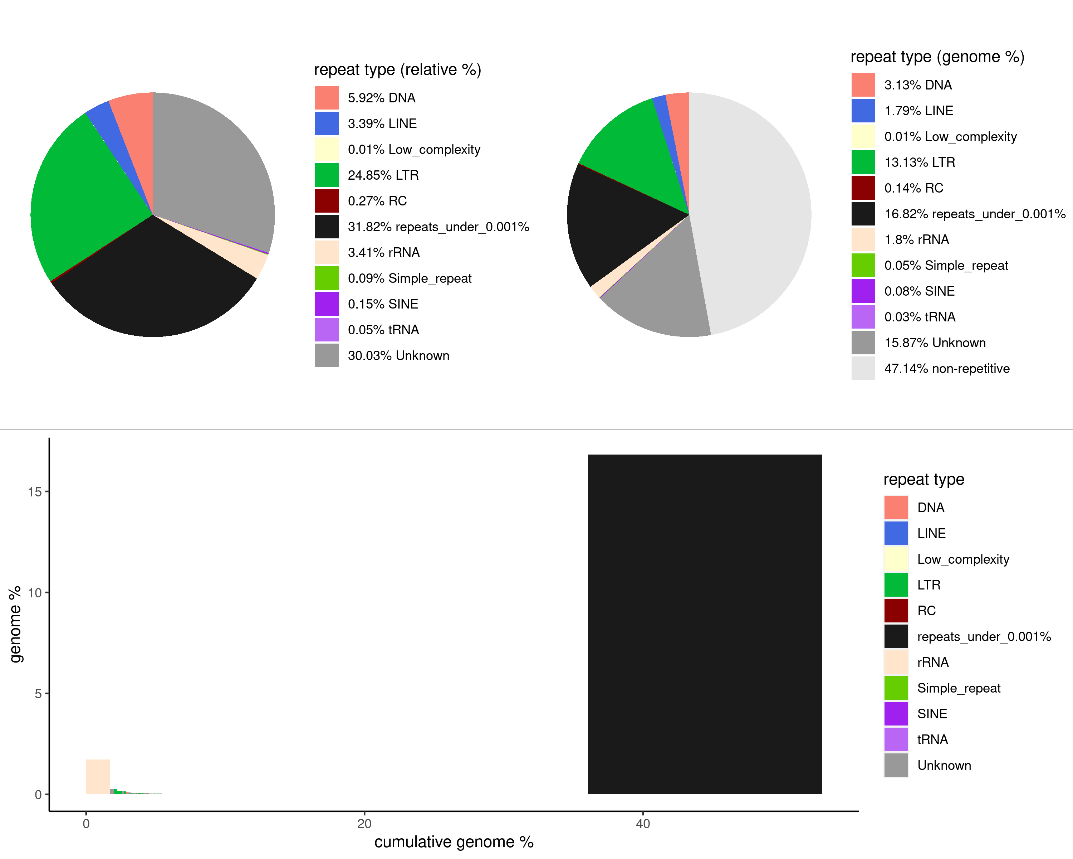


**Figure S7.** Proportion of repeats in subsampled *A. greggii* genome


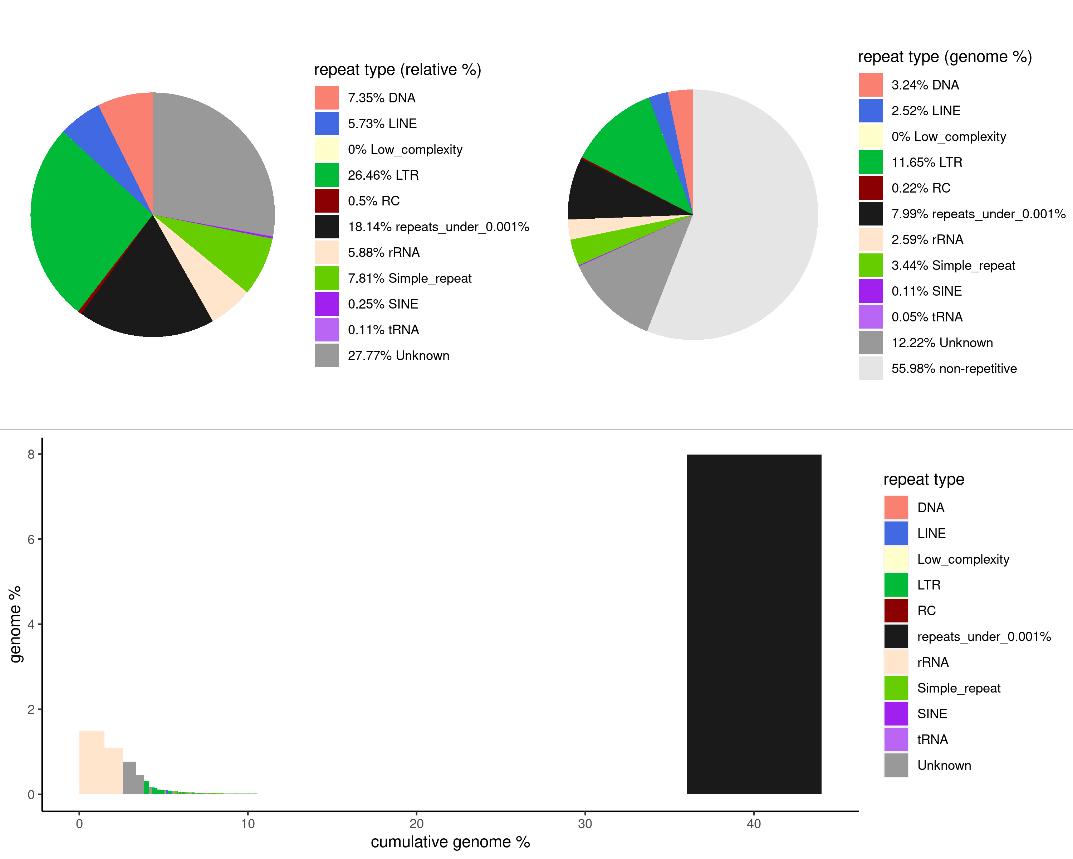


**Figure S8.** Proportion of repeats in subsampled *A. watsonii* genome


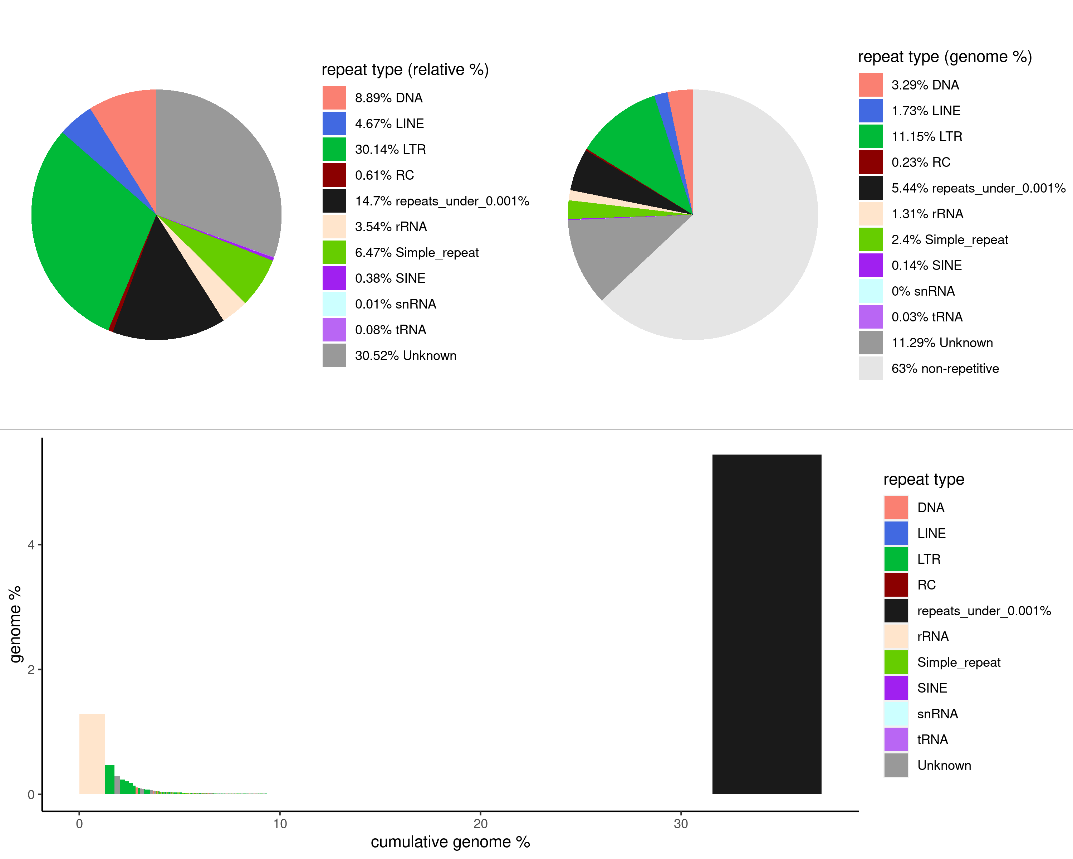


**Figure S9.** Proportion of repeats in subsampled *A. palmeri* genome


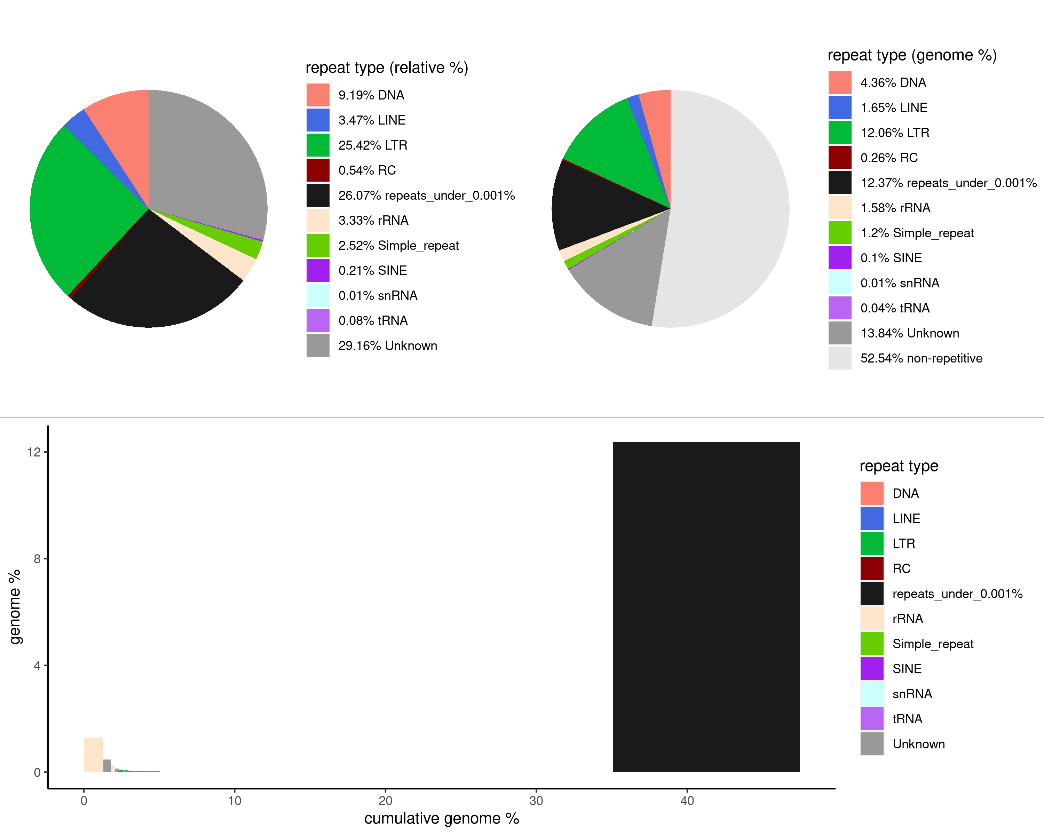


**Figure S10.** Proportion of repeats in subsampled *A. hybridus* genome


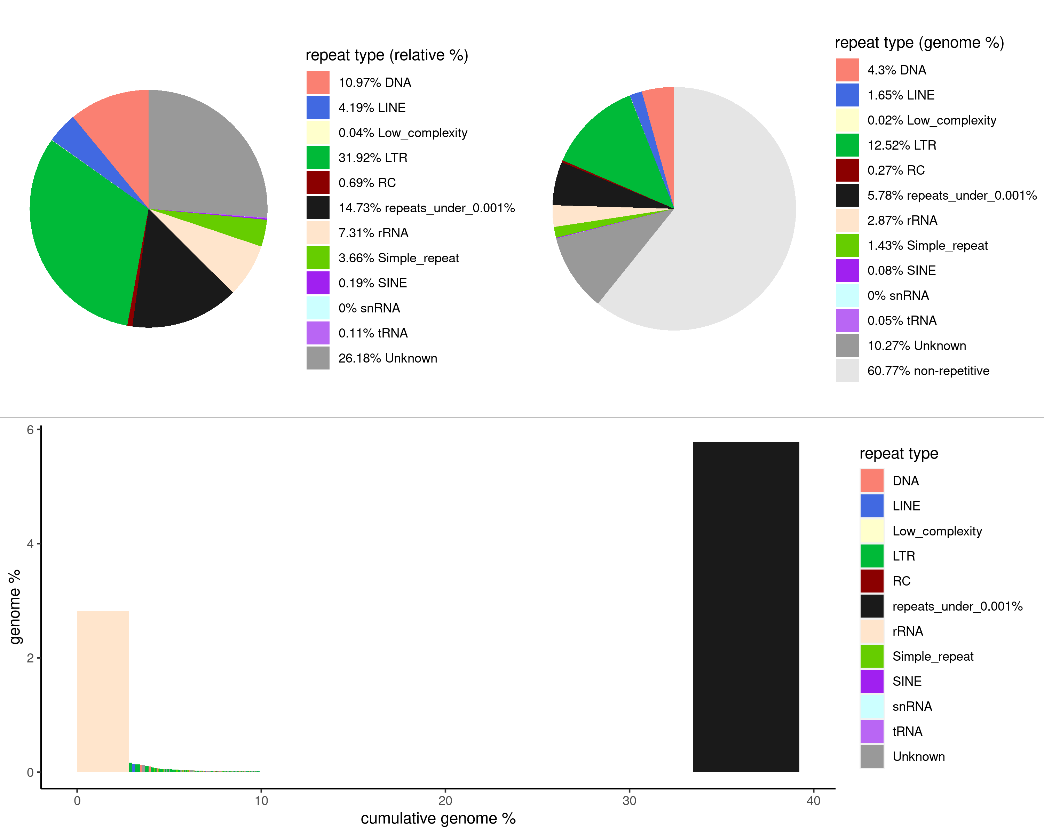


**Figure S11.** Proportion of repeats in subsampled *A. hypochondriacus* genome


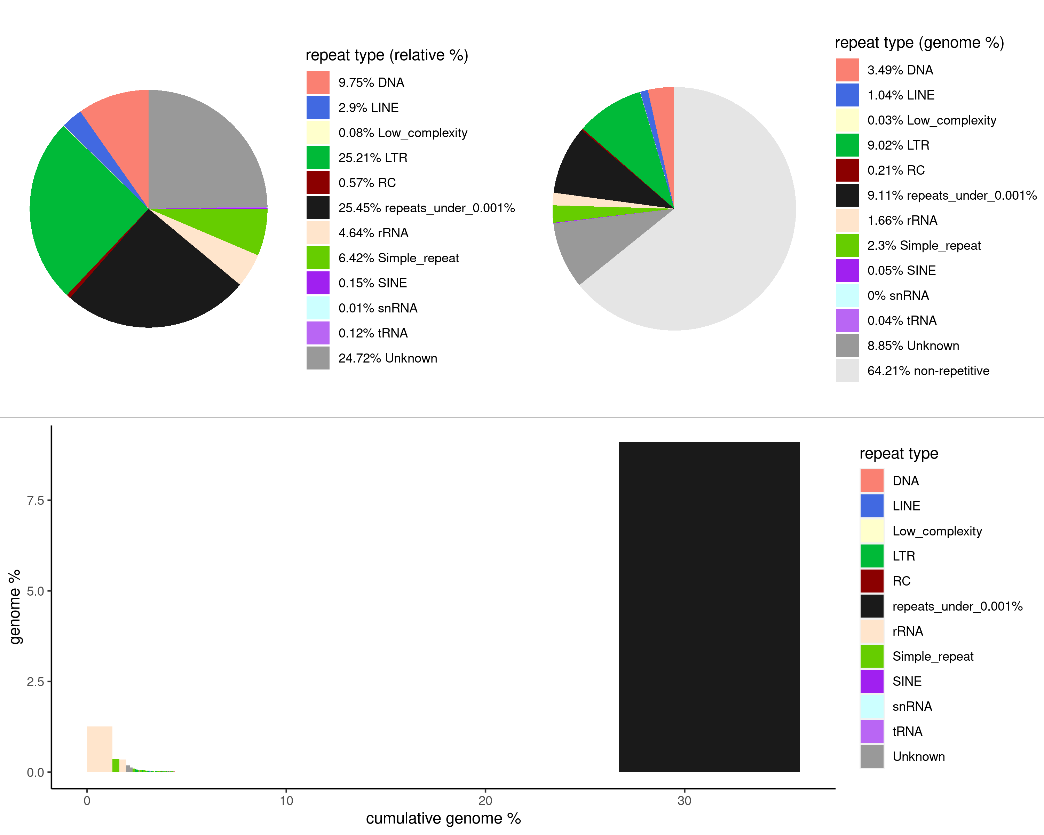


**Figure S12.** Proportion of repeats in subsampled *A. cruentus* genome
