# Supplementary material for: Genomic profiling of dioecious Amaranthus species provides novel insights into species relatedness and sex genes
Source: BMC Biol. 2023 Feb 20;21:37. doi: 10.1186/s12915-023-01539-9 (PMC9940365; doi:10.1186/s12915-023-01539-9)
Supplement: Supplementary file 6 — Additional file 6: Figures S1 – S3. Fig S1 – Upset plot of shared scaffolds with male- or female-enriched coverages. Fig S2 - Reads alignment coverage of male-to-female individuals for four dioecious Amaranthus species across scaffold 19. Fig S3 – Reads alignment coverage of male-to-female individuals for five dioecious Amaranthus species across FLOWERING LOCUS T (FT) on contig 00000542. [file 12915_2023_1539_MOESM6_ESM.docx]

**Additional file 6**


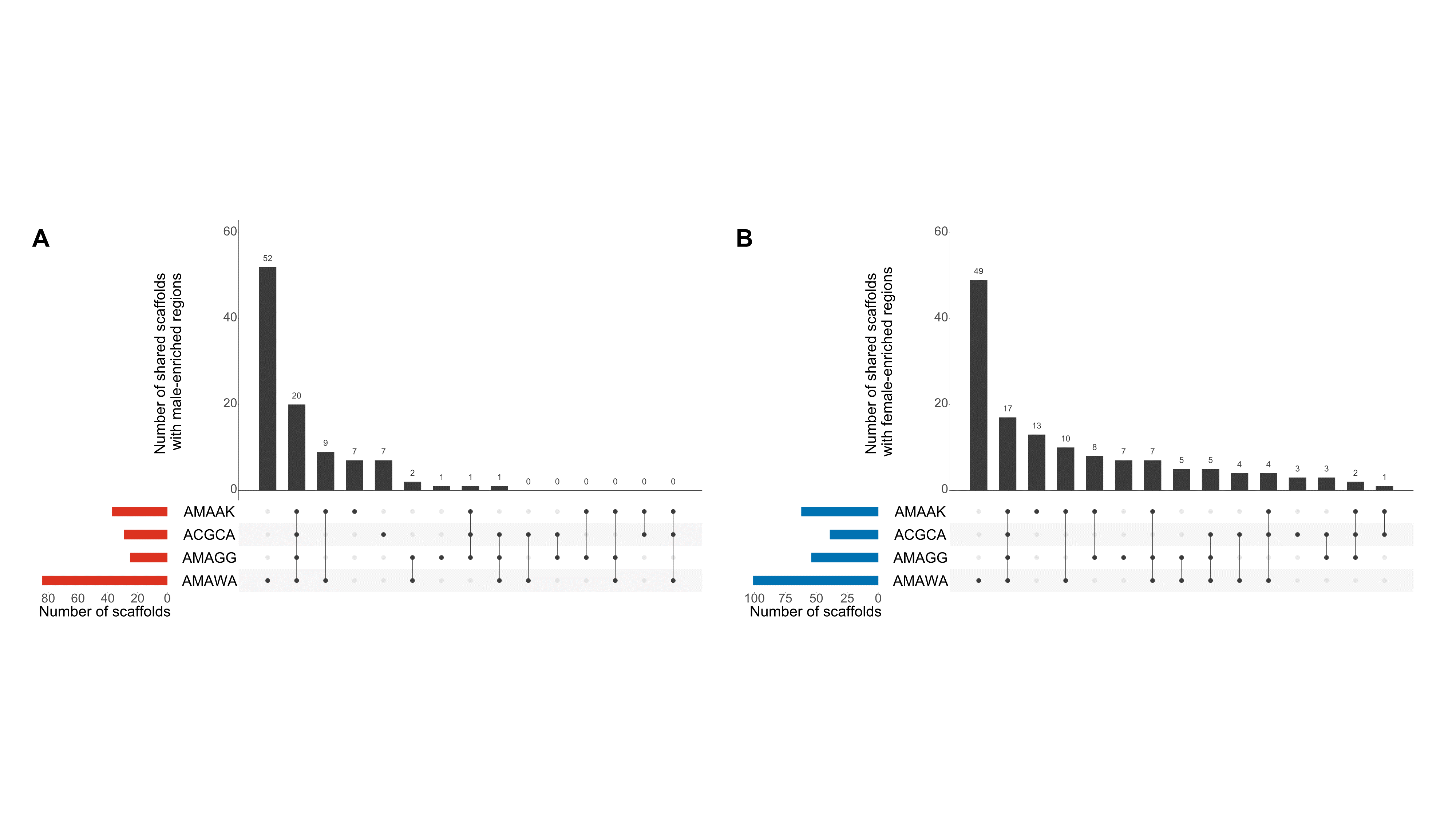


**Figure S1.** Upset plots delineating the number of shared scaffolds with male or female-enriched coverages. **A** Shared scaffold with regions enriched for male-specific coverages. **B** Shared scaffold with regions enriched for female-specific coverages. Species code represents the EPPO code for the four dioecious species: AMAAK (*Amaranthus acanthochiton*) ACGCA (*Amaranthus cannabinus*), AMAGG (*Amaranthus greggii*) and AMAWA (*Amaranthus watsonii*).


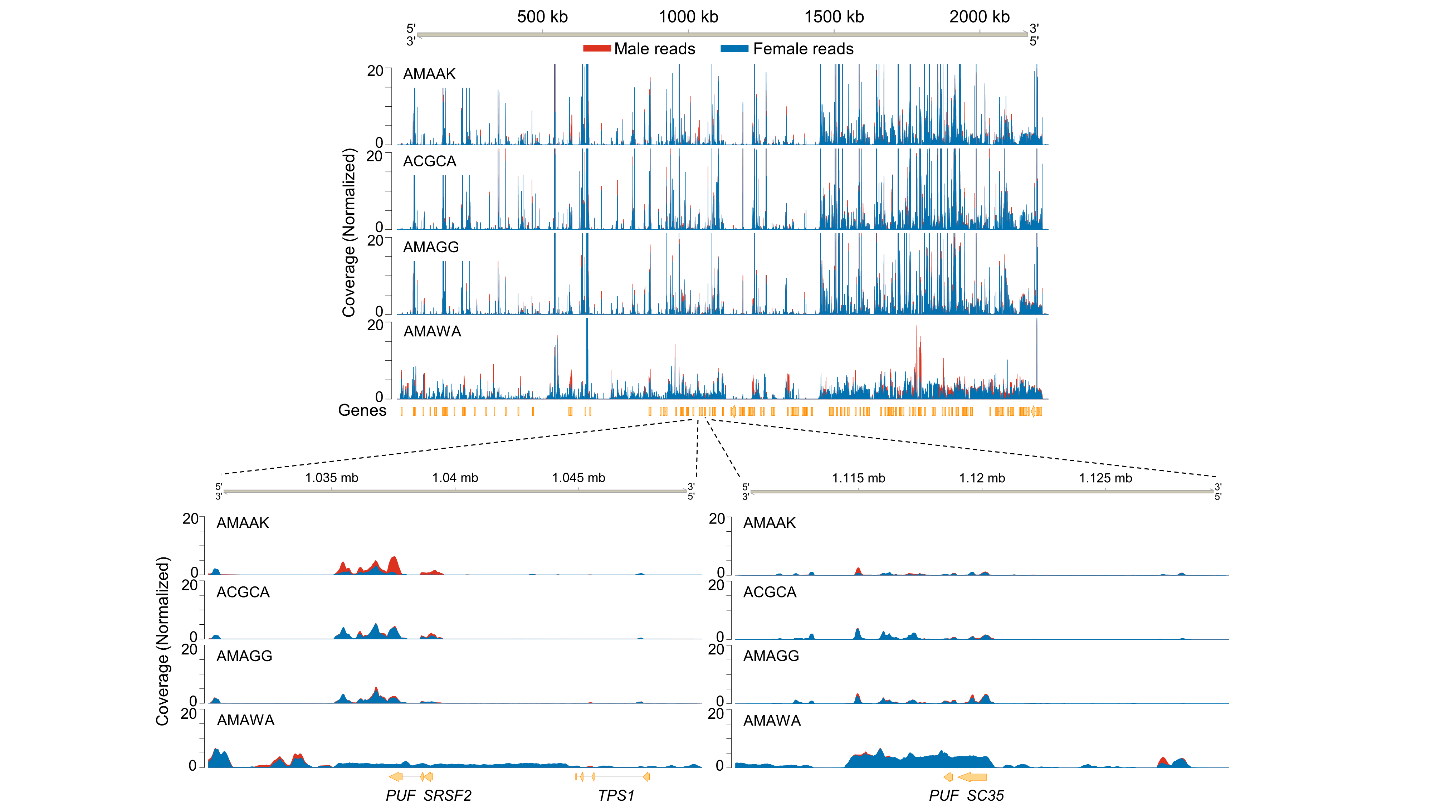


**Figure S2.** Reads alignment coverage of male-to-female individuals for four dioecious *Amaranthus* species across *A. palmeri* scaffold 19. Genes visualized within a 20-kb window.


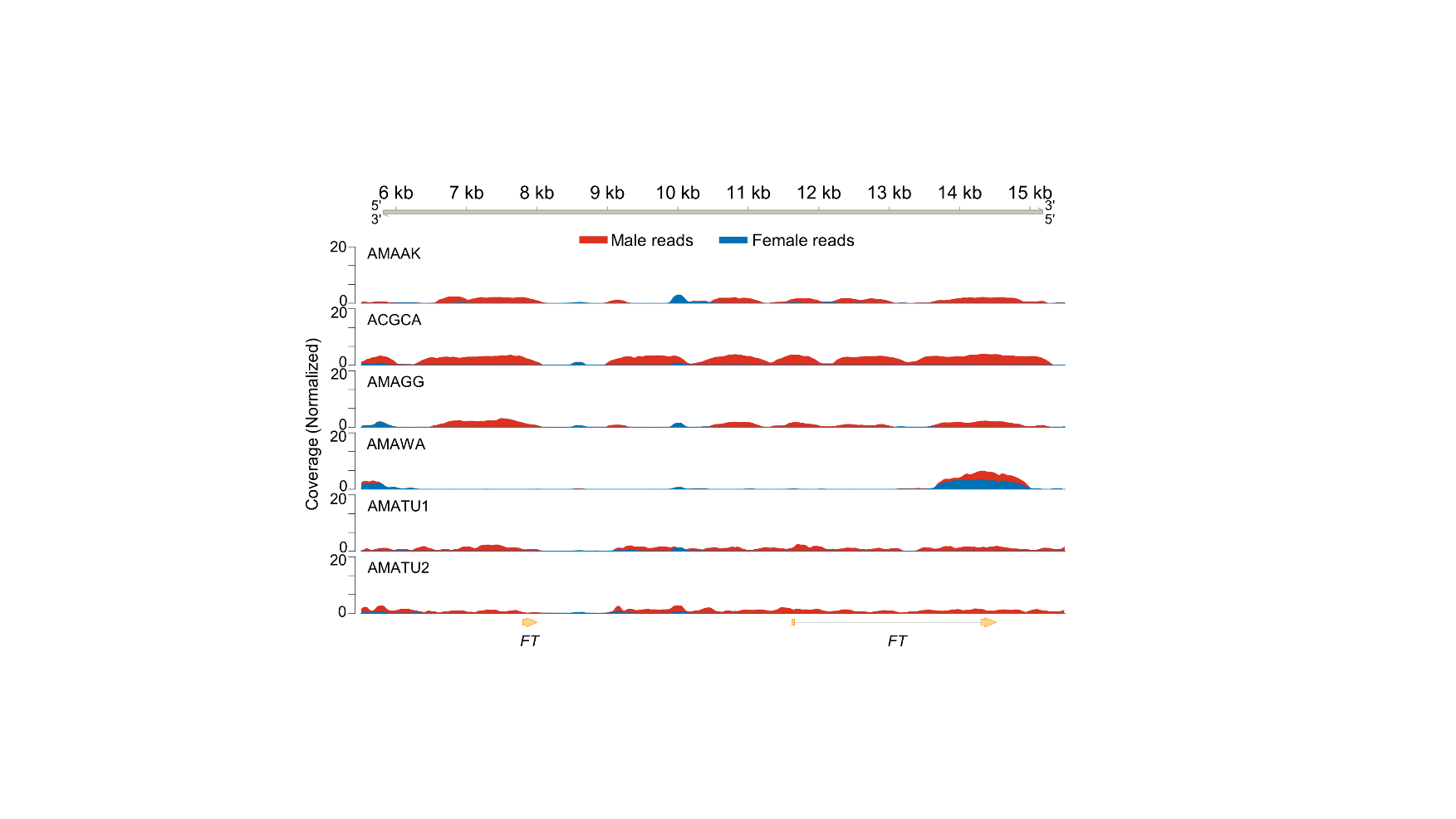


**Figure S3.** Reads alignment coverage of male-to-female individuals for five dioecious *Amaranthus* species across *A. tuberculatus FLOWERING LOCUS T* (*FT*) on contig 00000542. Species code AMATU1 (SRA numbers ERR3220246/ERR3220227) and AMATU2 (SRA numbers ERR3220310/ERR3220231) represent male/female individuals of *A. tuberculatus* from Kreiner et al. (2019). Genes visualized within a 10-kb window.
